# CD168 Identifies Proliferating Pancreatic Islet Cells in Murine and Human

**DOI:** 10.1002/advs.202510590

**Published:** 2025-12-21

**Authors:** Shubo Yuan, Jiafu Li, Min Shao, Haili Bao, Ajun Geng, Jialin Yang, Yu Tao, Xinyi Chen, Tianxiong Xiao, Chunye Liu, Zhiyao Xie, Wenqian Song, Qing Cissy Yu, Hongxing Fu, Xu Han, Taochen He, Wenquan Wang, Jianfeng Chen, Sheng Yan, Shaohua Song, Liang Liu, Yi Arial Zeng

**Affiliations:** ^1^ New Cornerstone Science Laboratory Key Laboratory of Multi‐Cell Systems Shanghai Institute of Biochemistry and Cell Biology Center for Excellence in Molecular Cell Science Chinese Academy of Sciences Shanghai 200031 China; ^2^ University of Chinese Academy of Sciences Beijing 100049 China; ^3^ Key Laboratory of Systems Health Science of Zhejiang Province School of Life Science Hangzhou Institute for Advanced Study Hangzhou 310024 China; ^4^ Frontier Innovation Center Department of Immunology School of Basic Medical Sciences Fudan University Shanghai 200032 China; ^5^ Department of Thoracic Surgery Shanghai Pulmonary Hospital Tongji University School of Medicine Shanghai 200433 China; ^6^ Department of General Surgery Ruijin Hospital School of Medicine Shanghai Jiaotong University Shanghai 200025 China; ^7^ Department of Hepatobiliary and Pancreatic Surgery of the Second Affiliated Hospital Zhejiang University School of Medicine Hangzhou Zhejiang 310003 China; ^8^ Department of Pancreatic Surgery Zhongshan Hospital Fudan University Shanghai 200032 China

**Keywords:** β‐cell maturation, β‐cell proliferation, CD168, cell surface marker, pancreatic islets

## Abstract

Restoring functional β‐cell mass through proliferation is a central goal of diabetes therapy, but progress has been hampered by the inability to isolate live, proliferating β‐cells for study. Here, CD168 is identified as a conserved surface marker that specifically enriches for proliferating cells in mouse and human pancreatic islets. Single‐cell RNA sequencing reveals a distinct cluster of islet cells with high proliferative activity, low insulin expression, and specific *CD168* expression. Flow cytometry and immunostaining confirm CD168^+^ cells co‐localize with Ki67^+^ proliferating cells, reside in G2/M phase, and comprise ≈0.5% of adult islet cells. Using a new *CD168‐CreERT2* mouse model for lineage tracing, it is demonstrated that CD168⁺ cells rapidly divide, forming two‐cell clones within hours. Unbiased lineage tracing shows 87.4% of clones are uni‐β lineage, with smaller proportions of uni‐α, uni‐δ, and multi‐lineage. Integrated lineage tracing with multi‐omics maps a ≈60‐day maturation trajectory for nascent β‐cells, involving progressive epigenome remodeling and shifting transcriptional networks. Notably, CD168 expression is conserved in proliferating cells of human islets and pancreatic neuroendocrine tumors, highlighting its clinical relevance. This work establishes CD168 as a marker for proliferating islet cells, providing a tool to study β‐cell proliferation and novel insights into islet cell proliferation and maturation mechanisms.

## Graphical abstract

This study identifies CD168 as a conserved surface marker for proliferating β‐cells in mouse, human islets, and pancreatic islet tumors. CD168⁺ cells show high proliferation and low insulin expression. CD168^+^ cells form mostly uni‐β lineage clones, and some of the clones are multi‐lineage. Multi‐omics and lineage tracing reveal a ≈60‐day maturation process for new β‐cells.

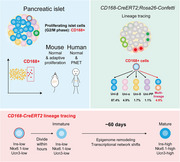

## 1 Introduction

Pancreatic islets, the endocrine compartment of the pancreas, orchestrate glucose homeostasis through tightly regulated hormone secretion. In mice, islets are predominantly composed of insulin‐producing β‐cells (≈80%), with smaller populations of α cells (glucagon), δ cells (somatostatin), and pancreatic polypeptide (PP) cells.^[^ ^1^ ^]^ β‐cells are indispensable for insulin production, and their functional mass is a critical determinant of metabolic health. Deficits in β‐cell mass, driven in part by their limited proliferative capacity, are central to diabetes pathogenesis.^[^ ^2^ ^]^ Restoring β‐cell mass through regeneration remains a pivotal therapeutic goal, yet progress has been stymied by an inability to isolate and characterize transiently proliferating β‐cell populations. The lack of specific surface markers to prospectively identify these cells has precluded molecular and functional analyses, underscoring a fundamental gap in diabetes research with profound implications for regenerative therapy development.

Notably, while β‐cell loss underpins diabetes, dysregulated islet cell proliferation can lead to pancreatic neuroendocrine tumors (PNETs),^[^ ^3^, ^4^ ^]^ which account for ≈12% of gastrointestinal neuroendocrine neoplasms, and exhibit rising incidence.^[^ ^5^ ^]^ This dual nature—balancing physiological regeneration against pathological hyperproliferation—highlights the need to define molecular mechanisms governing β‐cell proliferation in health and disease.

β‐cells display well‐documented functional and transcriptional heterogeneity, manifesting as variations in glucose responsiveness, insulin secretion, and regenerative potential.^[^ ^6^, ^7^, ^8^, ^9^, ^10^, ^11^, ^12^ ^]^ While markers such as CD81^high[^ ^13^ ^]^ and CD9^+^/ST8SIA1^+[^ ^12^, ^14^ ^]^ are enriched for immature subsets, markers like CD24,^[^ ^15^ ^]^ CD63,^[^ ^16^ ^]^ PDX1^high^ /MAFA^high^,^[^ ^17^ ^]^ Ucn3,^[^ ^18^ ^]^ Fltp^[^ ^10^ ^]^ and ERRγ^[^ ^19^ ^]^ define terminally differentiated mature β‐cells. However, critical gaps persist. First, the existing markers do not identify proliferative states or enrich for actively dividing subpopulations, which limits studies of β‐cell proliferation. Second, although it is hypothesized that immature β‐cells replenish mature pools, direct lineage‐tracing evidence for this transition is lacking. These limitations underscore the necessity for specific surface markers capable of isolating and tracing proliferating β‐cells throughout their maturation trajectory.

CD168 (Syndecan‐4/HMMR), a cell‐surface glycoprotein encoded by *Hmmr* (human: 5q33.2‐qter; mouse: Chr11), regulates proliferation, differentiation, and motility across diverse physiological and pathological contexts.^[^ ^20^, ^21^, ^22^, ^23^, ^24^ ^]^ Physiologically, CD168 marks replicating skeletal stem cells,^[^ ^25^, ^26^ ^]^ M2 macrophages,^[^ ^27^ ^]^ and hematopoietic progenitors.^[^ ^23^ ^]^ Pathologically, it drives metastasis in malignancies including breast, prostate, and colorectal cancers,^[^ ^28^, ^29^, ^30^, ^31^, ^32^, ^33^, ^34^, ^35^, ^36^ ^]^ yet its role in pancreatic islets—whether in normal proliferation or neoplasia—remains unexplored.

Here, we identify CD168 as a conserved surface marker of proliferative islet cells in mice and humans. By generating *CD168‐CreERT2* knock‐in mice for in vivo lineage tracing, we delineate the clonal dynamics and differentiation potential of CD168^+^ cells. Integrated multi‐omics profiling reveals dynamic epigenome remodeling and transcriptional network shifts during β‐cell maturation. Strikingly, CD168 expression extends to proliferating cells in human pancreatic islets and PNETs, linking physiological and pathological β‐cell proliferation. These findings bridge critical gaps in marker discovery, fate mapping, and molecular understanding, offering a cohesive framework to dissect islet cell proliferation, maturation trajectories, and molecular transitions in health and disease.

## 2 Results

### 2.1 Identification of a CD168⁺ Proliferative Subpopulation in Adult Murine Pancreatic Islets

**Figure 1:**
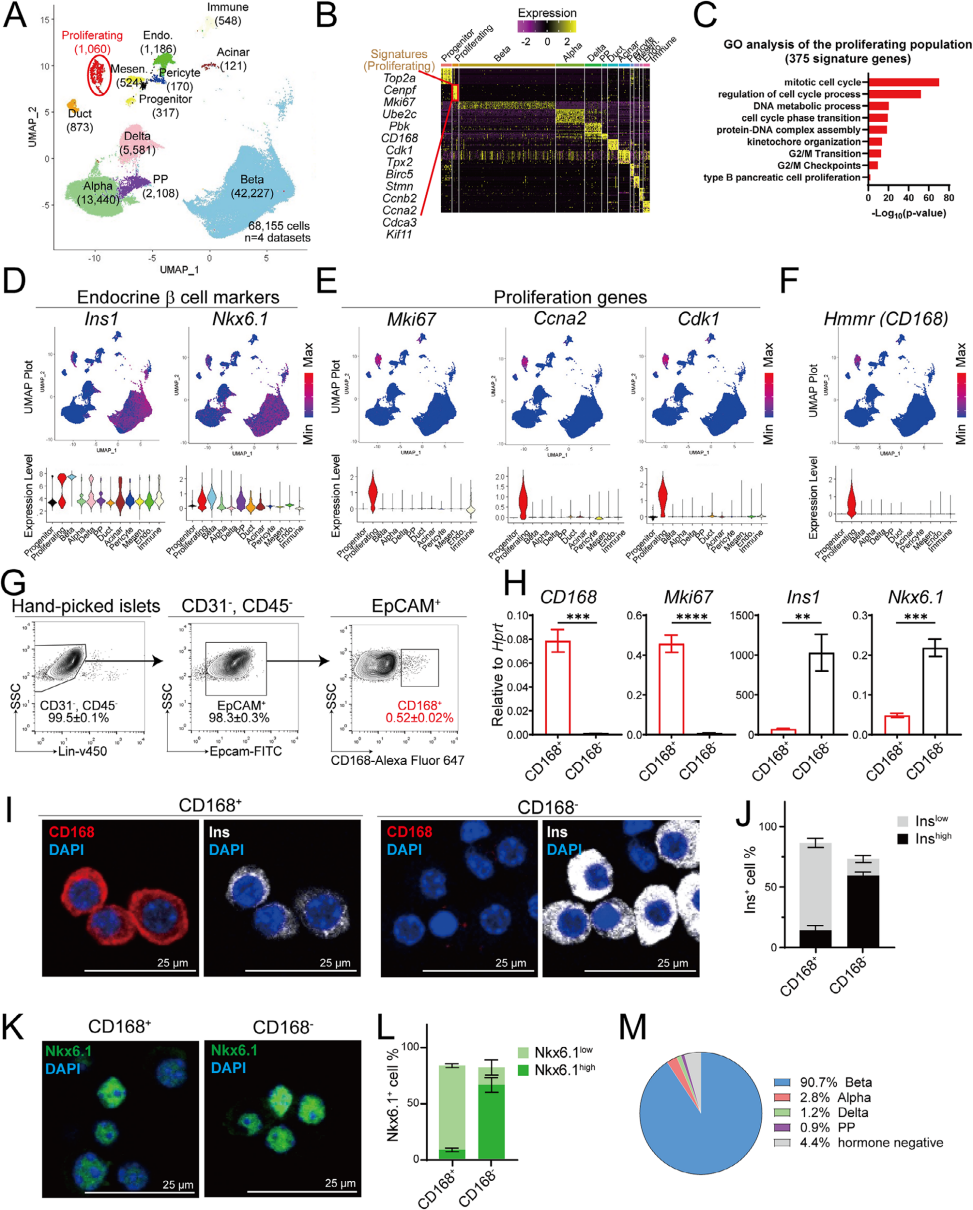
Identification of the CD168^+^ population that is enriched for proliferating islet cells with low Ins expression. A) UMAP plot of 68155 pancreatic single‐cell (sc) RNA‐seq profiles (points), colored by cluster assignment and annotated post hoc. The newly identified population (Proliferating) is circled in red. The number of cells is shown in brackets under the indicated cell type. Cells were pooled from n=4 datasets. Mensen., mesenchymal; Endo., endothelial. B) Heatmap of cell‐type‐enriched genes. Each column represents a single cell, and each row represents one signature gene. The colors ranging from purple to yellow indicate low to high relative gene expression levels. C) Selected GO terms of upregulated genes in the proliferating population. 375 genes were selected with min.pct =0.25 and logfc.threshold =0.25. D–F) Individual gene UMAP plots showing the expression levels and distribution of endocrine β (D) and proliferation (E) representative markers. The colors ranging from blue to red indicate low to high relative gene expression levels. The Vln plot shows the expression level (log2(TPM+1)) of the indicated genes in each cell type. The proliferating population expressed both proliferation markers and β‐cell markers, and expressed surface marker CD168 (F). G) FACS analysis of CD168‐expressing cells in hand‐picked mouse islets. The blood lineage cells (CD31 and CD45) were negatively selected, and epithelial cells (EpCAM) were positively selected. Within the CD31^−^CD45^−^EpCAM^+^ islet endocrine compartment, 0.52% ± 0.02% of cells were CD168^+^. Data were pooled from n=3 biological replicates and presented as mean ± SEM. H) qPCR analysis of *CD168*, *Mki67*, *Ins1*, and *Nkx6.1* of the proliferating population in FACS‐isolated cells. n=3 biological replicates. For each replicate, cells were isolated from 10 mice. Data are presented as mean ± SEM. An unpaired two‐tailed *t*‐test was used for comparison. ^**^ *p*<0.01, ^***^ *p*<0.001. I and J) Islet CD168^+^ and CD168^−^ cells were sorted by FACS for CD168/Ins immunostaining (I). Quantification indicating that the majority (70.5%) of CD168^+^ cells were Ins^low^, whereas 58.8% of CD168^−^ cells were Ins^high^ (J). Scale bar, 25 µm. Data were pooled from n=3 biological replicates and presented as mean ± SEM. K and L) Islet CD168^+^ and CD168^−^ cells were sorted by FACS for Nkx6.1 immunostaining (K). 73.1% of CD168^+^ cells were Nkx6.1^low^, while 64.2% CD168^−^ cells were Nkx6.1^high^ (L). Scale bar, 25 µm. Data were pooled from n=3 biological replicates and presented as mean ± SEM. M) Quantification indicating ≈90.7% CD168^+^ cells were Ins^+^, 2.8% CD168^+^ cells were Gcg^+^, 1.2% CD168^+^ cells were Sst^+^, 0.9% CD168^+^ cells were Ppy^+^, and 4.4% CD168^+^ cells were hormone negative.

To identify proliferating subpopulations, we performed single‐cell RNA sequencing analysis on endocrine‐enriched cells from postnatal and adult mice pancreas. We analyzed 68155 cells from our dataset^[^ ^37^ ^]^ and integrated published datasets^[^ ^13^, ^38^, ^39^ ^]^ (**Figure** **1**A; Figure S1A–D, Supporting Information). Clustering revealed populations of endocrine (α, β, δ, and PP) cells, exocrine (acinar and ductal), and other known populations (Procr^+^ progenitors, mesenchymal, endothelial, immune, and pericytes) (Figure 1A,B). Notably, we identified a unique cluster distinct from all known cell populations (Figure 1A). Gene Ontology (GO) analysis highlighted enrichment of mitotic cell processes, G2/M transition, and β‐cell (type B pancreatic cell) proliferation (Figure 1C).

This unique cluster expressed canonical proliferation markers (e.g., *Mki67*, *Cdk1*, *Top2a*, *Ccna2*) and displayed reduced expression of β‐cell identity genes (e.g., *Ins1*, *Nkx6.1*), as visualized by UMAP and violin plots (Figure 1B,D,E; Figure S1E,F, Supporting Information). The low Ins1 level, coupled with high expression of cell‐cycle regulators, supported a proliferative and immature phenotype.

To isolate this population, we screened for surface protein‐encoding genes within the cluster's signatures and identified CD168 as a specific marker (Figure 1F). We validated CD168's utility for live‐cell sorting by flow cytometry. After enzymatically digestion and dissociation of adult mouse islets into single cells, we excluded blood lineage cells (CD31^−^ CD45^−^) and enriched for epithelial cells (EpCAM⁺) before sorting CD168⁺ and CD168^−^ populations (Figure 1G). CD168⁺ cells constituted 0.52% ± 0.02% of adult islet cells (Figure 1G). RT‐qPCR confirmed the efficient separation, with CD168⁺ cells showing elevated *CD168* and *Ki67* expression and reduced Ins (insulin) expression compared to CD168^−^ cells (Figure 1H).

Immunostaining of sorted cells further validated that insulin and Nkx6.1 (mature β‐cell marker) protein levels were significantly lower in CD168⁺ cells (Figure 1I–L; Figure S1G, Supporting Information). Quantification showed that 90.7% of CD168^+^ cells were Ins^+^, 4.9% were positive for other hormones (2.8% Gcg^+^, 1.2% Sst^+^, and 0.8% Ppy^+^), and 4.4% were hormone‐negative (Figure 1M). Consistent with this, whole‐mount immunostaining of islets also detected CD168 expression in α, δ, and PP cells (Figure S1H—J, Supporting Information), which similarly appeared to have relatively lower hormone levels.

We next investigated the CD168 marker in diabetic conditions. In both Streptozotocin‐induced type 1 diabetes and high‐fat diet ‐induced type 2 diabetes mouse models, CD168^+^ β‐cells were Ki67^+^ and displayed low Ins levels in pancreas sections (Figure S2A,B, Supporting Information). Furthermore, we examined the response of CD168^+^ cells to the reported proliferative stimuli—including Harmine,^[^ ^40^, ^41^ ^]^ Exendin‐4 (Ex4),^[^ ^42^, ^43^, ^44^ ^]^ and high glucose.^[^ ^45^ ^]^ In vitro culture of isolated islets demonstrated that these stimuli increased the proportion of CD168^+^ cells (Figure S2C–G, Supporting Information). Thus, we identify CD168 as a surface marker that enriches for a proliferative, immature cell state present in homeostatic and diabetic conditions.

### 2.2 CD168 Marks Proliferating Cells in G2/M Phase in Pancreatic Islets

**Figure 2:**
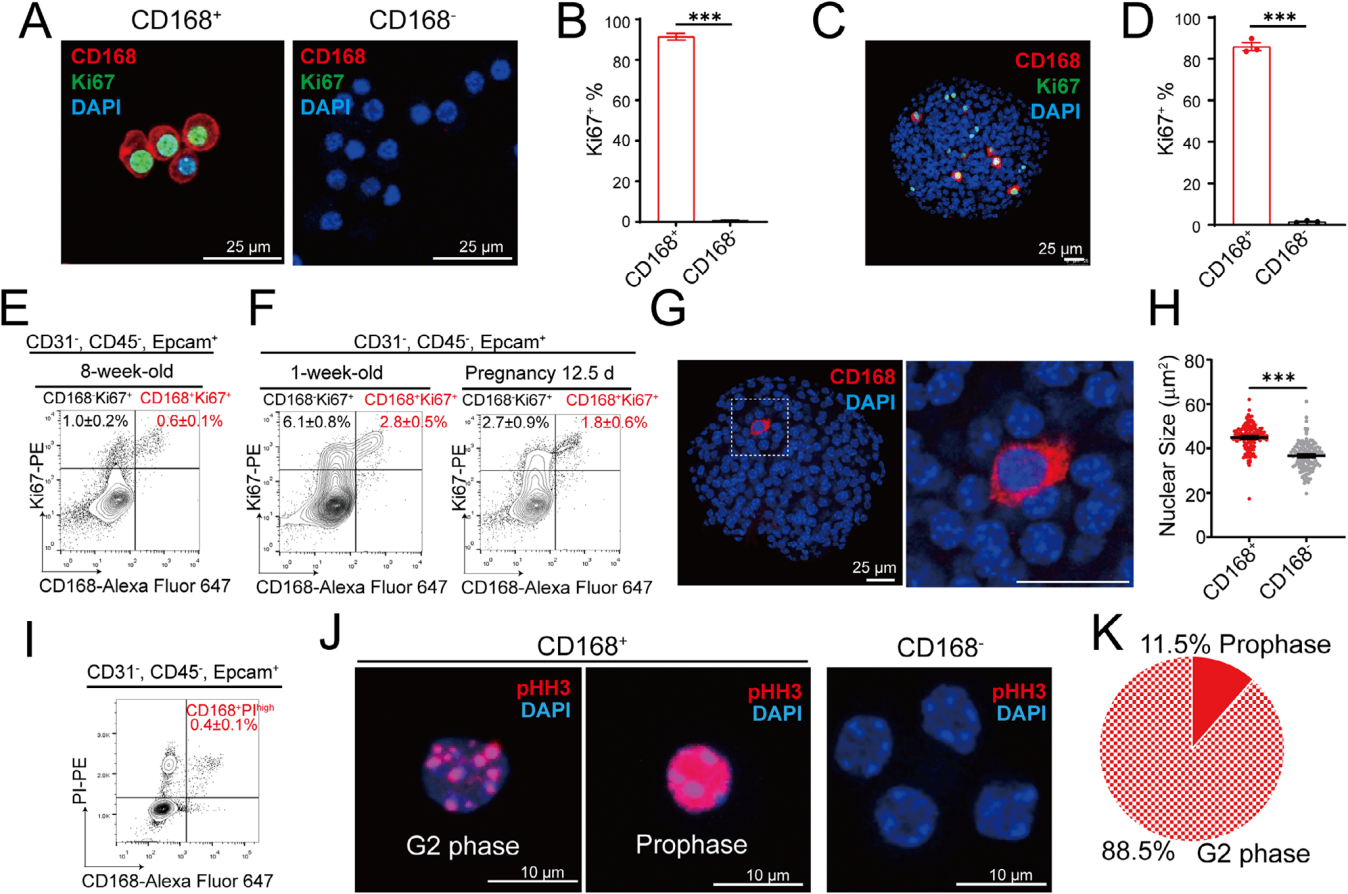
CD168 marks proliferating cells in G2/M phase in pancreatic islets. A,B) Islet CD168^+^ and CD168^−^ cells were sorted by FACS for CD168/Ki67 immunostaining, showing that CD168^+^ cells expressed proliferation marker Ki67 (A). Quantification indicating that 91.4% of CD168^+^ cells were Ki67^+^, while 0.5% of CD168^−^ cells were Ki67^+^ (B). Scale bar, 25 µm. Similar results were confirmed by n=3 mice. An unpaired two‐tailed *t*‐test was used for comparison. ^***^ *p*<0.001. C and D) Representative confocal image of an 8‐week‐old mouse islet whole mount immunostaining, indicating that CD168^+^ cells expressed Ki67 in situ (C). Scale bar, 25 µm. Quantification indicating that 85.7% of CD168^+^ cells were Ki67^+^, while 0.9% of CD168^−^ cells were Ki67^+^ (D). Similar results were confirmed by n=3 mice. An unpaired two‐tailed *t*‐test was used for comparison. ^***^ *p*<0.001. E) FACS analysis of CD168 and Ki67 co‐staining in fixed handpicked islet cells from 8‐week‐old mice. The majority of CD168^+^ cells were Ki67^+^, and ≈37.5% Ki67^+^ cells were CD168^+^. Data were pooled from n=3 biological replicates and presented as mean ± SEM. F) The left panel shows newborn 1‐week‐old islet cells, and the right panel shows pregnant day 12.5 islet cells. Data were pooled from n=3 biological replicates and presented as mean ± SEM. G and H) Representative confocal image of an 8‐week‐old mouse islet whole mount immunostaining, indicating that the nuclear size of CD168^+^ cells was larger in situ (G). Scale bar, 25 µm. Quantification indicated that CD168^+^ cells exhibited a larger nuclear size than CD168^−^ cells. (H). Similar results were confirmed by n=3 mice. An unpaired two‐tailed *t*‐test was used for comparison. ^***^ *p*<0.001. I) FACS analysis of CD168 and PI co‐staining in fixed handpicked islet cells. The majority of CD168^+^ cells were PI^high^. Data were pooled from n=3 biological replicates and presented as mean ± SEM. J and K) Islet CD168^+^ and CD168^−^ cells were sorted by FACS for pHH3 immunostaining, showing that CD168^+^ cells expressed proliferation marker pHH3 (J). The left panel showed that pHH3 was punctate, indicating the cell was at the G2 phase. The middle panel showed that pHH3 smeared in the nucleus, indicating the cell was at mitotic prophase (J). Quantification indicating that 88.5% of CD168^+^ cells were at G2 phase, and 11.5% of CD168^+^ cells were at prophase (K). Scale bar, 25 µm. Similar results were confirmed by n=3 mice.

To define the cell‐cycle position of CD168⁺ cells, we first assessed their proliferative status. Immunostaining revealed that 91.4 ± 1.7% of sorted CD168⁺ cells were Ki67⁺ (**Figures** **2**A,B; S1G, Supporting Information). This was confirmed in intact islets (Figure 2C,D). Notably, not all Ki67^+^ cells were CD168^+^, indicating CD168 marks a specific proliferative subset (Figure 2C). Flow cytometry quantification supported this, showing that the vast majority of CD168^+^ cells were Ki67^+^; they constituted only a fraction (37.5%) of the total Ki67⁺ pool in adult islets (0.6 % CD168^+^ Ki67^+^ vs 1.6% Ki67^+^) (Figure 2E).

As expected, the overall proliferation rate was higher in physiologically proliferative states. The percentage of Ki67⁺ cells was elevated in postnatal day 7 (1‐week‐old) islets (≈8.9%) and in islets from pregnant mice at day 12.5 (≈4.7%) (Figure 2F). The proportion of CD168⁺ cells increased correspondingly at these stages (2.8% at P7 and 1.8% at pregnancy day 12.5), with the ratio of CD168⁺Ki67⁺ cells to total Ki67⁺ cells remaining consistent (Figure 2E,F).

We next sought to pinpoint their specific cell‐cycle phase. CD168⁺ cells exhibited a larger nuclear size (Figure 2G,H), suggesting increased DNA content.^[^ ^46^ ^]^ Propidium iodide (PI) staining by flow cytometry confirmed that CD168⁺ cells possessed higher DNA content, consistent with a 4N DNA complement (Figure 2I). Finally, immunostaining for phospho‐histone H3 (Ser10) (pHH3) demonstrated that CD168⁺ cells were predominantly in the G2 (punctate nuclear pHH3, 88.5%) and M (full nuclear pHH3, 11.5%) phases of the cell cycle (Figure 2J,K).^[^ ^47^, ^48^ ^]^ Collectively, these data demonstrate that CD168 specifically enriches for a subpopulation of islet cells that are actively progressing through the G2/M phase of the cell cycle.

### 2.3 Lineage Tracing of CD168^+^ Cells in Adult Pancreatic Islets and Rapidly Renewing Tissues

**Figure 3:**
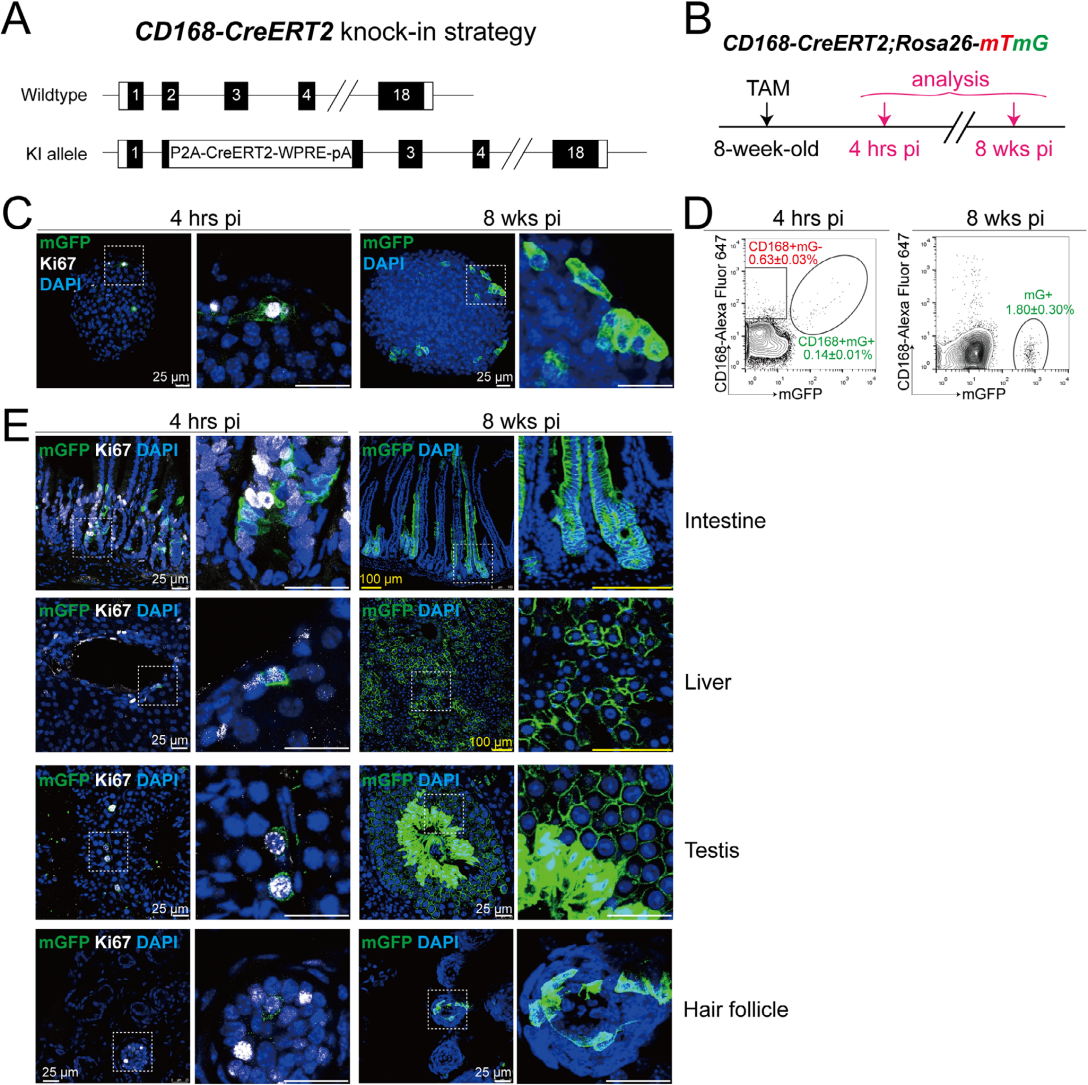
Lineage tracing of CD168^+^ cells in pancreatic islets and various tissues. A) Targeting strategy to generate the *CD168‐CreERT2* knock‐in (KI) mouse. B) Illustration of the lineage tracing strategy of CD168^+^ cells using *Rosa26‐mTmG* reporter. 8‐week‐old mice were administered with tamoxifen (TAM) (4 mg/25 g body weight), and tissues were harvested after 4 h or 8 weeks post‐induction. C) The left two panels show representative confocal images of the islet whole mount from *CD168‐CreERT2;Rosa26‐mTmG* mice at 4 h, indicating that at the beginning of tracing (4 h), mGFP^+^ labeled cells were Ki67^+^. The right two panels show representative confocal images of the islet whole mount from *CD168‐CreERT2;Rosa26‐mTmG* mice 8 weeks after TAM treatment. n=3 mice. Scale bars: 25 µm. D) Flow cytometry analysis and quantification of mGFP^+^ cell percentage in the islets at 4 hrs pi (left) and 8 wks pi (right). At 4 hrs pi, 0.14% of islet cells were labeled with mGFP, and all mGFP^+^ cells were CD168^+^. At 8 wks pi, the mGFP^+^ cell percentage increased to 1.8%. Data were pooled from n=3 biological replicates and presented as mean ± SEM. E) Representative confocal images of intestine, liver, testis, and hair follicle frozen section from *CD168‐CreERT2;Rosa26‐mTmG* mice at 4 hrs pi, indicating that at the beginning of tracing, mGFP^+^ labeled cells were Ki67^+^ (Left two panels). The right two panels show representative confocal images at 8 wks pi. n=3 mice. Scale bars: yellow, 100 µm; white, 25 µm.

To track the division and fate of CD168^+^ islet cells, we generated a *CD168‐CreERT2* knock‐in mouse model using CRISPR‐Cas9. A CreERT2 cassette was inserted into exon 2 of the *CD168* locus, ensuring CreERT2 expression under the control of the endogenous promoter (**Figure** **3**A; Figure S3A–C, Supporting Information). Heterozygous mice were grossly normal and showed no detectable defects in islet morphology or glucose homeostasis compared to wild‐type littermates (Figure S3D–G, Supporting Information).

For lineage tracing, we crossed *CD168‐CreERT2* mice with *Rosa26‐mTmG* reporter mice.^[^ ^49^ ^]^ Adult (8‐week‐old) *CD168‐CreERT2*;*Rosa26‐mTmG* mice received a single tamoxifen (TAM) pulse, and islets were analyzed at short‐term (4 h) and long‐term (8 weeks) post‐induction (pi) (Figure 3B). At 4 h pi, whole‐mount imaging revealed sparse, membrane‐localized GFP^+^ (mGFP^+^) cells that universally co‐expressed Ki67 (Figure 3C). Flow cytometry confirmed that mGFP⁺ cells (0.14% of islet cells) were exclusively CD168⁺, demonstrating specific labeling (Figure 3D). The labeling efficiency was ≈18% (0.14% mGFP^+^, CD168^+^ cells vs 0.77% CD168^+^ cells).

By 8 weeks pi, we observed a marked expansion of mGFP^+^ clones, predominantly localized at the islet periphery (Figure 3C). Flow cytometry indicated mGFP⁺ cells increased to 1.8% of islet cells (Figure 3D). This marked expansion from 0.14% to 1.8%, despite the low proliferative activity of adult islets, demonstrates the robust clonal expansion of CD168⁺ cell‐derived progeny. Furthermore, these progeny were largely CD168^−^, consistent with their differentiation into post‐mitotic, mature cells.

To evaluate the broader utility of our model, we also assessed CD168⁺ cells in rapidly turning‐over tissues, including the small intestine, liver, testis, and hair follicle. At 4 h pi, individual mGFP⁺ cells co‐localized with Ki67⁺ proliferating cells (Figure 3E). By 8 weeks pi, these had expanded into extensive mGFP⁺ clones (Figure 3E), confirming *CD168‐CreERT2* as a versatile tool for lineage tracing of proliferating populations across diverse tissues.

### 2.4 Clonal Analysis Reveals Unipotent and Multipotent CD168⁺ Cells

**Figure 4:**
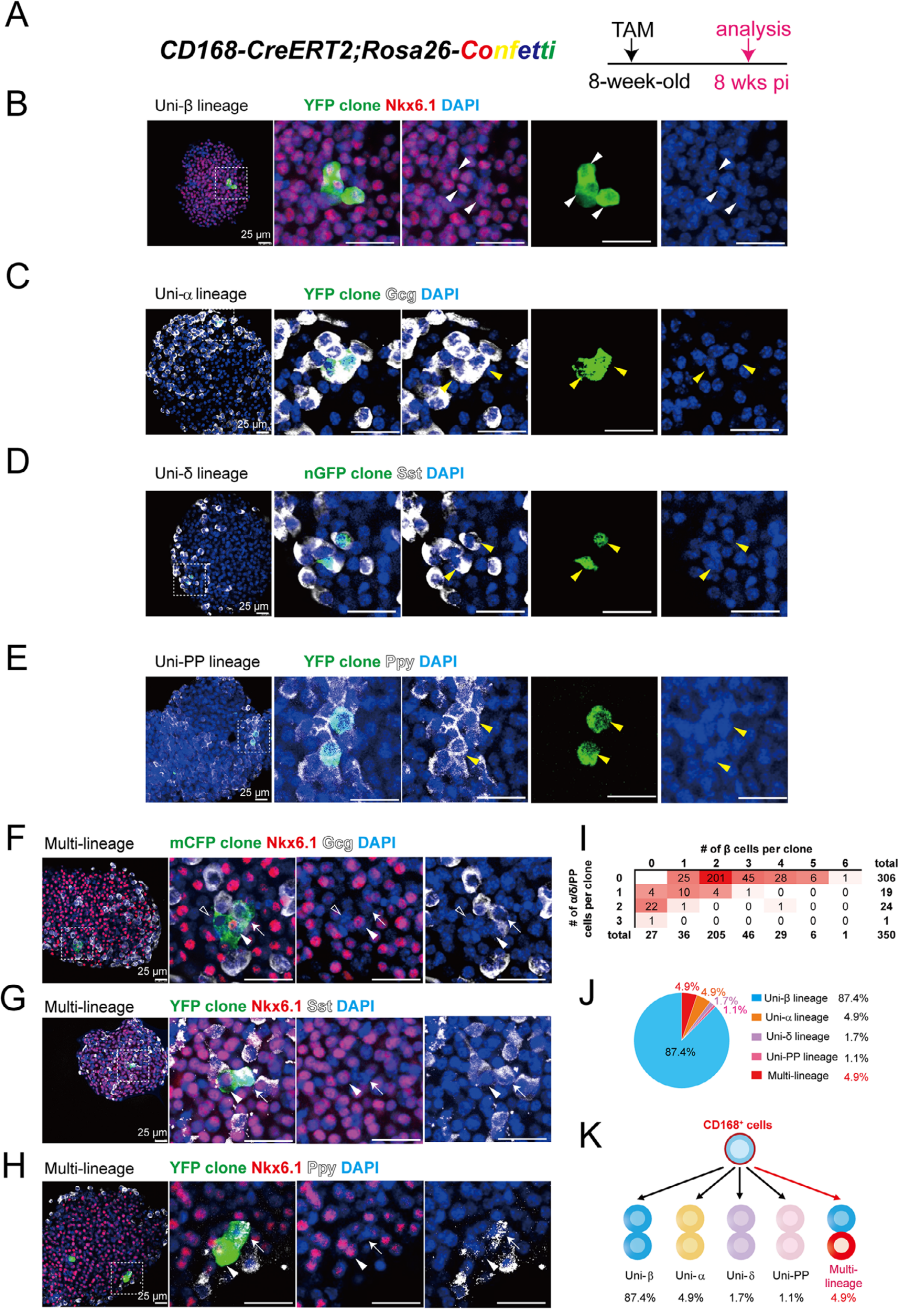
Clonal analysis reveals unipotent and multipotent CD168^+^ cells. A) Illustration of the lineage tracing strategy of CD168^+^ cells using *Rosa26‐Confetti* reporter in adult islets. 8‐week‐old mice were administered TAM (2 mg/25 g body weight), and tissues were harvested after 8 weeks. B–E) Representative whole mount immunostaining of islets demonstrating the existence of uni‐β lineage (white solid arrowhead) (B), uni‐α lineage (yellow solid arrowhead) (C), uni‐δ lineage (yellow solid arrowhead) (D), and uni‐PP lineage (yellow solid arrowhead) (E). n=8 mice. Scale bar, 25 µm. F–H) Representative whole mount immunostaining of islets demonstrating the existence of multi‐lineage, including β (solid arrowhead) + α (arrow) + potential hormone negative cell (open arrowhead) clone (F), β (solid arrowhead) + δ (arrow) clone (G), and β (solid arrowhead) + PP (arrow) clone (H). n=8 mice. Scale bar, 25 µm. I) Clonal analysis at 8 weeks pi. β‐cell numbers are shown along the *x*‐axis, and α, δ, or PP cell numbers are shown along the *y*‐axis. Red shading indicates the relative frequency of certain clone compositions, with deeper shading indicating higher frequency. n=8 mice, 350 clones. J) Quantification indicating that 4.8% of clones were multi‐lineage, while 87.4% of clones were uni‐β lineage, 4.9% of clones were uni‐α lineage, 1.7% of clones were uni‐δ lineage, and 1.1% of clones were uni‐PP lineage. n=8 mice, 350 clones. K) Illustration of the CD168^+^ cell lineage model.

To resolve the developmental potential of CD168⁺ cells at a clonal level, we crossed *CD168‐CreERT2* mice with *Rosa26‐Confetti* reporters^[^ ^50^ ^]^ and analyzed islets 8 weeks post‐tamoxifen induction (**Figure** **4**A). Unbiased lineage tracing revealed that majority of clones were unipotent: 87.4% gave rise exclusively to Nkx6.1^high^ mature β‐cells (uni‐β; Figure 4B,J), 4.9% to glucagon⁺ α cells (uni‐α; Figure 4C,J), 1.7% to somatostatin⁺ δ cells (uni‐δ; Figure 4D,J), and 1.1% to pancreatic polypeptide⁺ (PP) cells (uni‐PP; Figure 4E,J).

Notably, 4.9% of clones were multipotent, contributing to at least two endocrine lineages. These included β/α (Figure 4F), β/δ (Figure 4G), and β/PP (Figure 4H) combinations. The predominance of two‐cell clones suggests that most labeled CD168⁺ cells underwent only one or two divisions during the 8‐week tracing period (Figure 4I; Figure S3H, Supporting Information).

Given our previous identification of multipotent Procr⁺ progenitors,^[^ ^37^ ^]^ we investigated a potential overlap. Using the *Procr‐mGFP‐2A‐lacZ* mouse model, we identified a rare population of proliferating Procr^+^CD168^+^Ki67^+^ cells with islets (Figure S3I, Supporting Information). Flow cytometry quantified this population at 5.1% of total CD168⁺ cells (Figure S3J, Supporting Information), a proportion strikingly similar to the frequency of multipotent clones (4.9%).

We next asked whether this fate potential is retained in a physiological state of β‐cell expansion. We performed lineage tracing during pregnancy, administering TAM at day 12 and analyzing islets one week post‐induction (Figure S3K, Supporting Information). Under these conditions, clonal output was overwhelmingly unipotent, with 96.6% of CD168⁺ cell‐derived clones contributing to the β‐cell lineage alone (Figure S3L–O, Supporting Information). This aligns with the established model that direct β‐cell replication is the primary driver of islet expansion during pregnancy.^[^ ^51^, ^52^ ^]^ In summary, these data demonstrate that the CD168⁺ population in adult islets is functionally heterogeneous, composed predominantly of unipotent endocrine progenitors alongside a rare subset of multipotent progenitors, the latter of which may be marked by Procr expression.

### 2.5 Lineage Tracing Documents the Time Course of β‐Cell Maturation

**Figure 5:**
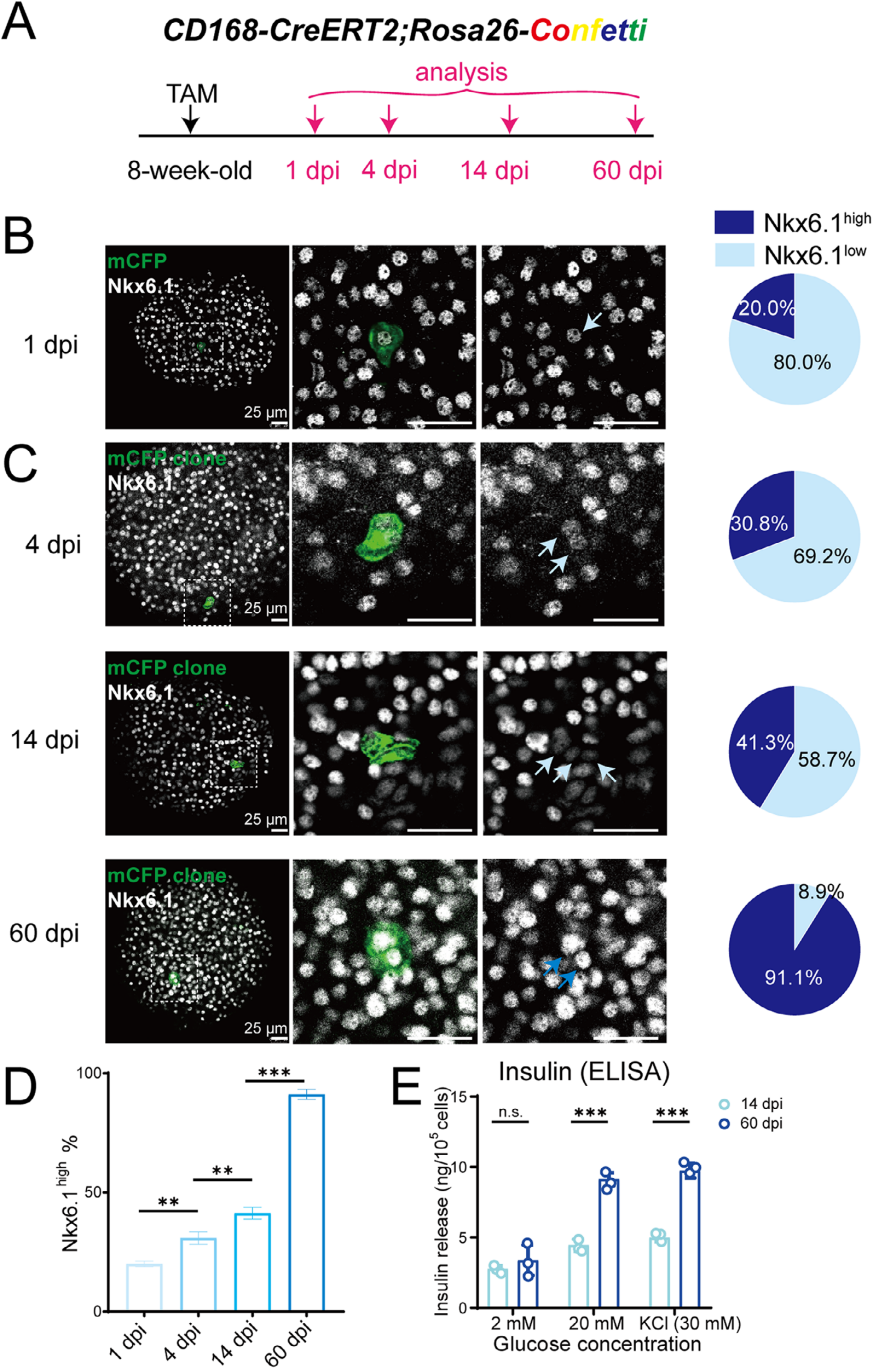
Lineage tracing documents the time course of β‐cell maturation. A) Illustration of the lineage tracing strategy of CD168^+^ cells using *Rosa26‐Confetti* reporter in adult islets. 8‐week‐old mice were administered with TAM (2 mg/25 g body weight) and tissues were harvested after 1 day, 4 days, 14 days, or 60 days. B) Representative islet whole mount immunostaining of mCFP and Nkx6.1 (left) and quantification (right) indicating that at the beginning of tracing (1 dpi), 80% of labeled cells were Nkx6.1^low^, while 20% were Nkx6.1^high^, n=3 mice, 105 labeled cells. Scale bar, 25 µm. C and D) Representative whole mount immunostaining of islets (C), indicating that the proportion of Nkx6.1^high^ in traced cells increased to 30.8% at 4 dpi, to 41.3% at 14 dpi, and to 91.1% at 60 dpi. The sky‐blue arrows indicate Nkx6.1^low^ cells, and the dark‐blue arrows indicate Nkx6.1^high^ cells. n=3 mice, >100 labeled cells in each group. Scale bar, 25 µm. Quantification of the percentage of Nkx6.1^high^ cells in traced clones (D). ^**^ *p*<0.01, ^***^ *p*<0.001. E) GSIS experiments using sorted *CD168‐CreERT2;Rosa26‐mTmG* traced cells, verifying that 14 dpi traced cells were less mature than 60 dpi traced cells. n.s., not significant. ^***^ *p*<0.001.

While β‐cells are known to undergo a slow maturation process,^[^ ^53^, ^54^ ^]^ the precise timeline and functional progression of this process in vivo remain poorly defined. To map the maturation trajectory of nascent β‐cells, we leveraged the *CD168‐CreERT2* model to trace newly divided cells, the majority of which adopt β‐cell fate. Adult *CD168‐CreERT2;Rosa26‐Confetti* mice received a tamoxifen pulse, and clones were analyzed at 1, 4, 14, and 60 days post‐induction (dpi) (**Figure** **5**A).

At 1 dpi, the vast majority (91.3%) of labeled cells were still single, and 80.0% exhibited low Nkx6.1 expression, confirming their immature state (Figure 5B). This finding is consistent with our transcriptomic and immunostaining data from sorted CD168⁺ cells (Figure 1). By 4 dpi, most labeled cells (67.6%) had divided to form 2‐cell clones; however, 69.2% of the progeny still maintained low Nkx6.1 levels, indicating that maturation had not yet commenced (Figure 5C). At 14 dpi, clones were still predominantly 2‐cell (87.5%), but the proportion of Nkx6.1^high^ cells increased significantly from 30.8% (at 4 dpi) to 41.3%, demonstrating the onset of progressive maturation (Figure 5D). Finally, by 60 dpi, maturation was nearly complete, with 91.1% of labeled cells expressing high levels of Nkx6.1 (Figure 5D).

To functionally validate this timeline, we performed glucose‐stimulated insulin secretion (GSIS) assays on traced cells sorted at 14 and 60 dpi (Figure 5E; Figure S3K, Supporting Information). Consistent with the marker expression, cells at 60 dpi exhibited significantly enhanced insulin secretion compared to those at 14 dpi, confirming their acquisition of mature β‐cell function (Figure 5E). Together, these results provide an integrated timeline of postnatal β‐cell maturation, demonstrating that newly divided CD168⁺ progeny require ≈2 months to fully acquire the mature molecular and functional profile of established β‐cells.

### 2.6 Epigenome Remodeling and Shifting Transcription Networks During β‐Cell Maturation

**Figure 6:**
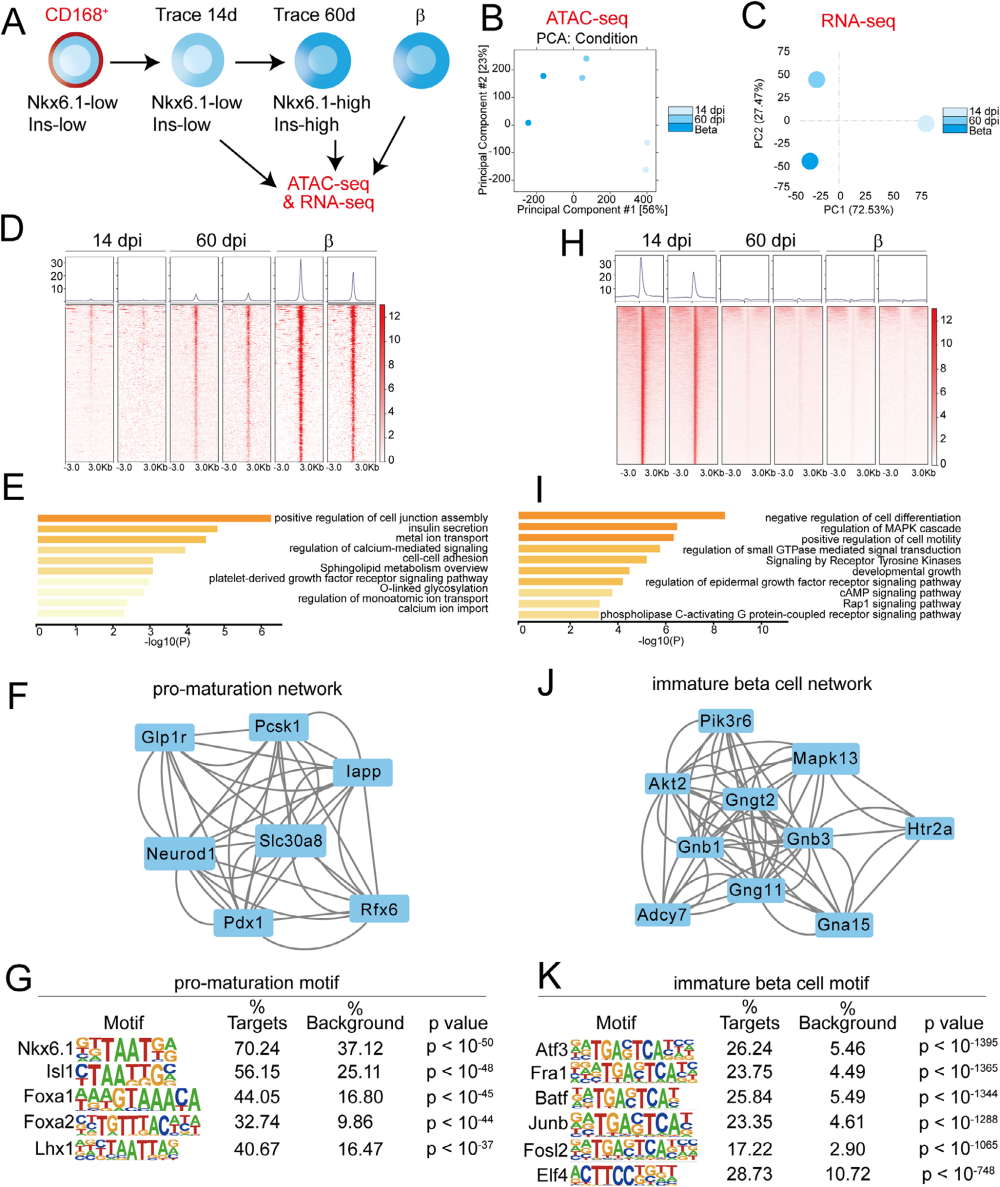
ATAC‐seq reveals epigenome remodeling and shift of transcription network during β‐cell maturation. A) Assay for transposase‐accessible chromatin with high‐throughput sequencing (ATAC‐seq) and RNA‐seq were performed using sorted *CD168‐CreERT2;Rosa26‐mTmG* traced cells at 14 dpi and 60 dpi, and mature β‐cells. B) Principal Component Analysis (PCA) of 14 dpi, 60 dpi, and β‐cells in ATAC‐seq. C) PCA of 14 dpi, 60 dpi, and β‐cells in RNA‐seq. D‐G) The difference analysis of peaks showed that 504 loci gradually opened during 14 dpi to 60 dpi (D). GO analysis of the 504 loci (E). Transcriptional factor (TF) network of the pro‐maturation 504 loci (F). The pro‐maturation TF motif (G). H–K) The difference analysis of peaks showed that 15865 loci specifically opened in 14 dpi (H). GO analysis of the 15865 loci (I). TF network of the immature β‐cell 15865 loci (J). The immature β‐cell TF motif (K).

To define the regulatory dynamics underlying β‐cell maturation, we performed ATAC‐seq and bulk RNA‐seq on FACS‐isolated, traced cells at 14 dpi, 60 dpi, and on mature β‐cells (**Figure** **6**A; Figure S4A, Supporting Information). Both transcriptomic and epigenomic profiles showed that 60 dpi cells exhibited greater transcriptional and epigenomic similarity to mature β‐cells than 14 dpi cells, confirming their progression toward a mature state (Figure 6B,C).

We identified 504 genomic loci that progressively gained chromatin accessibility over the 60‐day maturation period (Figure 6D). These opening regions were enriched near genes critical for insulin secretion (e.g., *Ins2*, *Mafa*, *Pcsk1*; Figure S4B, Supporting Information), whose expression increased concomitantly (Figure S4C, Supporting Information). Pathway analysis linked these regions to essential maturation processes, including cell junction organization,^[^ ^55^ ^]^ sphingolipid metabolism,^[^ ^56^ ^]^ chemical synaptic transmission, and metal ion transport (Figure 6E). Notably, we also identified two less‐characterized pathways: PDGFR signaling—previously implicated in development^[^ ^57^ ^]^ and O‐linked glycosylation, a post‐translational modification that may regulate membrane receptor activity during maturation^[^ ^58^ ^]^ (Figure 6E).

Protein‐protein interaction (PPI) network analysis of these open chromatin loci revealed a transcriptional hub centered on the key β‐cell regulators Pdx1 and Neurod1 (Figure 6F). Motif enrichment within these accessible regions confirmed the binding sites for essential β‐cell transcription factors like Nkx6.1, Isl1, and Foxa1/2, and revealed the dynamic activation of Lhx1 regulatory elements (Figure 6G). RNA‐seq confirmed the elevated expression of these factors (*Nkx6.1*, *Isl1*, *Foxa2*, *Lhx1*) in 60 dpi and mature β‐cells (Figure S4D, Supporting Information).

Conversely, we identified 15865 loci that progressively lost accessibility, representing regions active in immature cells but silenced upon maturation (Figure 6H). These closing regions are associated with pathways that negatively regulate differentiation and promote proliferation, such as the MAPK cascade and receptor tyrosine kinase signaling (Figure 6I). PPI networks analysis of highlighted nodes centered on Mapk13 and Akt2 (Figure 6J). Motif enrichment identified known regulators of immaturity, including Atf3 for glucose tolerance^[^ ^59^ ^]^ and Junb for insulin gene repression,^[^ ^59^ ^]^ along with four unreported stage‐specific regulators (Fra1, Fosl2, Batf, Elf4; Figure 6K). Their elevated expression in 14 dpi cells was confirmed by RNA‐seq (Figure S4C, Supporting Information).

Collectively, these multi‐omics data delineate a coordinated regulatory program for β‐cell maturation, driven by the activation of maturation‐promoting genes through chromatin opening and the simultaneous silencing of proliferative and differentiation‐inhibitory pathways through chromatin closing.

### 2.7 CD168 Marks Proliferating Cells in Human Islets and PNETs

**Figure 7:**
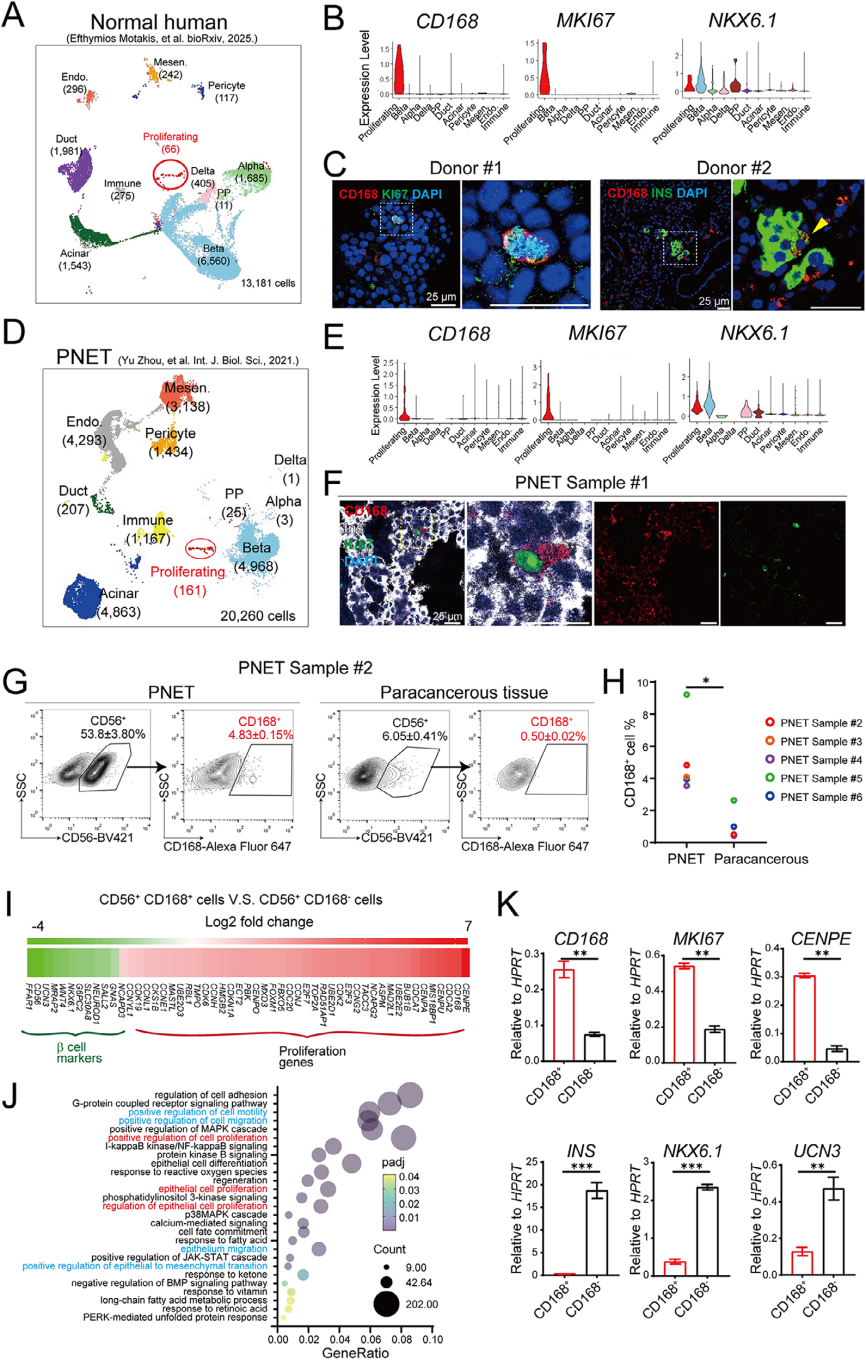
CD168 is a conserved proliferating cell surface marker in human PNETs. A) UMAP plot of normal human pancreas scRNA‐seq profiles (points), colored by cluster assignment and annotated post hoc. The new population (Proliferating) is circled in red. The number of cells is shown in brackets under the indicated cell type. Cells were pooled from published data. Mensen., mesenchymal; Endo., endothelial. B) The Vln plot shows the expression level (log2(TPM+1)) of the indicated gene in each cell type. The proliferating population expressed higher proliferation markers and lower β‐cell markers. C) Representative confocal image of a normal adult human pancreas sample frozen section, indicating that CD168^+^ cells were KI67^+^ (left two panels) and INS^low^ (right two panels). Scale bar, 25 µm. D) UMAP plot of human PNET scRNA‐seq profiles (points), colored by cluster assignment and annotated post hoc. The new population (Proliferating) is circled in red. The number of cells is shown in brackets under the indicated cell type. Cells were pooled from published data. Mensen., mesenchymal; Endo., endothelial. E) The Vln plot shows the expression level (log2(TPM+1)) of the indicated gene in each cell type. The proliferating population expressed both proliferation markers and β‐cell markers. F) Representative confocal image of adult human PNET sample frozen section, indicating that CD168^+^ cells were KI67^+^ and INS^low^. Scale bar, 25 µm. G) FACS analysis of CD168‐expressing cells in human PNET sample and paracancerous tissue. The blood lineage (CD31 and CD45)^+^ and non‐islet cells (CD56^−^) were excluded. Within the CD31^−^CD45^−^CD56^+^ islet endocrine compartment, the proportion of CD168^+^ cells was higher in the PNET sample. Data were presented as mean ± SEM. H) Quantification of CD168^+^ cell percentage in 5 PNET samples. An unpaired two‐tailed *t*‐test was used for comparison. ^*^ *p*<0.05. I) RNA‐seq analysis indicating upregulation of proliferation‐related genes and downregulation of mature β‐cell‐related genes in CD168^+^ cells compared to CD168^−^ cells. J) GO analysis of CD168^+^ cells enriched proliferating pathways. K) RT‐qPCR analysis of proliferating markers *CD168*, *MKI67*, and *CENPE*, and mature β‐cell markers *INS*, *NKX6.1*, and *UCN3* in FACS‐isolated cells as indicated. n=3 biological replicates. For each replicate, cells were isolated from 1 human PNET sample. Data were presented as mean ± SEM. An unpaired two‐tailed *t*‐test was used for comparison. ^**^ *p*<0.01, ^***^ *p*<0.001.

To determine if the CD168⁺ proliferative population is conserved in humans, we first analyzed a published single‐cell RNA sequencing dataset of healthy human pancreases.^[^ ^60^ ^]^ We identified a small population of CD168⁺ cells (**Figure** **7**A) that, mirroring our murine data, displayed high expression of the proliferation marker *MKI67* and reduced expression of mature β‐cell markers like *NKX6.1* (Figure 7B). We confirmed the presence of these CD168⁺ cells in pancreatic tissue sections from two healthy human organ donors (Figure 7C).

We next investigated CD168 in the context of human PNETs, which account for 1–2% of pancreatic neoplasms.^[^ ^61^, ^62^ ^]^ Analysis of a human PNET scRNA‐seq dataset^[^ ^61^ ^]^ revealed a distinct proliferative subpopulation that specifically expressed *CD168* (Figure 7D,E), This neoplastic CD168⁺ population shared key characteristics with its murine and healthy human counterparts, exhibiting elevated *MKI67* and reduced *NKX6.1* expression (Figure 7E). Immunohistochemistry on PNET patient samples confirmed the co‐localization of CD168 and KI67 (Figure 7F).

To quantify this proliferative compartment, we used flow cytometry, combining CD168 with CD56 (NCAM), a known endocrine cell marker and prognostic marker in PNETs.^[^ ^63^, ^64^, ^65^ ^]^ PNET tissues contained a significantly higher CD168⁺ cell frequency of CD168⁺ cells than adjacent peritumor non‐neoplastic tissues (4.85 % vs 0.50%; Figure 7G). This increase was consistent across all five patients analyzed, with CD168⁺ cells constituting 3.5–9.2% of PNET cells compared to only 0.4–2.6 % in control tissues (Figure 7H).

Transcriptomic analysis of sorted CD168⁺ PNET cells revealed an enrichment of proliferation‐associated genes (e.g., *CENPE*, *CENPA*, *E2F3*, *TOP2A*, *CDK6*) and a depletion of differentiation markers (e.g., *NEUROD1*, *NKX6.1*, *UCN3*) (Figure 7I). KEGG pathway analysis further highlighted enrichment in processes linked to tumor aggressiveness, including cell motility, MAPK signaling, NF‐κB activation, and epithelial proliferation (Figure 7J). These findings were validated by RT‐qPCR, with CD168⁺ cells showing elevated *CD168*, *MKI67*, and *CENPE* expression, alongside reduced *INS*, *NKX6.1*, and *UCN3* levels (Figure 7K). Collectively, these results establish CD168 as a conserved marker of proliferating endocrine cells in both mouse and human, in both physiological and neoplastic conditions.

## 3 Discussion

Therapeutic strategies for diabetes aim to enhance functional β‐cell mass, yet progress has been hampered by the inability to isolate and study the rare, proliferating β‐cell fraction. In this study, we identify the surface protein CD168 as a specific marker for proliferating islet cells, addressing this critical technical gap. We show that CD168⁺ cells in murine and human islets are proliferative and transcriptionally immature, and we establish the conservation of this population in human PNETs. The identification of CD168 provides a foundational tool to isolate live proliferating endocrine cells, opening new avenues for exploring the mechanisms of β‐cell replication, maturation, and tumorigenesis.

While the present study does not directly interrogate the molecular function of CD168, our finding that it specifically marks islet cells in the G2/M phase is strongly supported by its established role in cell cycle progression. A substantial body of evidence positions CD168 as a pivotal regulator of mitosis. It is a centrosomal protein required for spindle pole stability through interactions with Dynein,^[^ ^66^ ^]^ and its expression is tightly regulated, peaking at the G2/M phase.^[^ ^67^, ^68^ ^]^ Functionally, CD168 is essential for the activation of Aurora Kinase A, a master mitotic regulator; its silencing disrupts spindle assembly and delays mitotic progression.^[^ ^69^, ^70^, ^71^, ^72^ ^]^ Furthermore, CD168 promotes cell cycle transitions by regulating key cyclins like Cyclin B1, and its loss impairs proliferation in various cell types.^[^ ^30^, ^73^, ^74^ ^]^ This conserved role in facilitating mitosis provides a plausible mechanistic basis for why CD168 robustly identifies the proliferative fraction in both normal islets and PNETs. The well‐documented overexpression of CD168 across diverse carcinomas^[^ ^75^, ^76^, ^77^, ^78^, ^79^, ^80^, ^81^, ^82^, ^83^, ^84^, ^85^, ^86^, ^87^, ^88^, ^89^, ^90^, ^91^, ^92^ ^]^ and its association with poor prognosis further underscore its fundamental link to proliferation and highlight its potential clinical relevance beyond the pancreas.

By integrating *CD168‐CreERT2* lineage tracing with multi‐omics approaches, we delineated a maturation timeline of nascent β‐cells, a process governed by coordinated epigenetic and transcriptional reprogramming. Our analysis revealed that maturation involves a dual regulatory mechanism: the progressive opening of chromatin near key β‐cell identity genes, such as *Pdx1* and *Neurod1*, correlates with their increasing expression, and the simultaneous closure of loci. Conversely, loci that were enriched for pro‐proliferation factors and differentiation inhibitors, including *Atf3*, *Junb*, *Fra1*, *Fosl2*, *Batf*, and *Elf4*. The dynamic opposition between opening loci (promoting maturity) and closing loci (turning off proliferation) creates a “chromatin clock” that times the acquisition of β‐cell function. The identification of novel pathways within the opening chromatin regions, including PDGFR signaling and O‐linked glycosylation, which were previously unassociated with β‐cell maturation, expands the known mechanistic repertoire of maturation and presents new potential targets for enhancing functional maturation in vitro or therapeutic intervention.

The functional outcome of this reprogramming was a gradual acquisition of metabolic competence, as confirmed by GSIS assays showing that CD168⁺‐derived progeny required ≈60 days to reach full functional maturity. The precise inheritance of these progressive epigenetic states through cell division is likely critical for ensuring that daughter cells commit to a mature β‐cell fate. While its specific role remains to be defined, the specific expression of CD168 during the G2/M phase positions it as a potential facilitator in the faithful transmission of this maturation program to subsequent generations.

Our lineage‐tracing data provide new insights into the post‐mitotic maturation of β‐cells, which may help clarify the nature of previously identified immature populations. We observed that the progeny of CD168⁺ cells frequently localize to the islet periphery and exhibit an immature molecular profile (Ucn3^low^), a signature that aligns with the “virgin β‐cell” state described in earlier work.^[^ ^53^, ^93^ ^]^ The prior conclusion that these virgin β‐cells represent a stable lineage was based on the constant proportion of Ucn3^−^ cells within a broadly labeled β‐cell pool using* Ins‐CreER*.^[^ ^53^ ^]^ This finding could be interpreted to suggest a lack of conversion from the immature to the mature state. However, an alternative explanation is that this static ratio reflects a dynamic equilibrium, where the rate of new immature cells being generated through division is balanced by their rate of maturation.

Our results lend support to this dynamic model. By specifically tracing from a proliferative and immature state, we were able to capture the gradual maturation of these cells over a ≈60‐day period. This leads us to a plausible hypothesis: the virgin β‐cell state may be a transient, post‐mitotic phase through which newly divided cells pass. In this model, proliferation of existing β‐cells—whether from a mature or immature progenitor—could transiently yield Ucn3^−^ progeny that subsequently undergo slow maturation to replenish the functional pool. This would maintain the relative proportions of Ucn3⁺ and Ucn3^−^ cells observed in static snapshots, even as individual cells transition between states. Thus, while not contradicting the descriptive findings of earlier studies, our direct tracing from the CD168⁺ compartment suggests that the virgin β‐cell phenotype is part of a maturation continuum rather than a terminal fate, offering a revised framework for understanding β‐cell population dynamics.

Our lineage‐tracing experiments reveal that the CD168⁺ proliferative compartment in adult islets is not uni‐lineage fate‐restricted. Although most clones were unipotent β‐cells (87.4%), we consistently observed a subset of multi‐lineage clones (4.9%) that generated β‐ and α‐, δ‐, or PP‐cell progeny. These data indicate that unipotent β‐cell proliferation coexists with a small residual pool of multipotent progenitors under homeostatic conditions. The presence of multipotent CD168⁺ cells aligns with reports of plastic or progenitor‐like cells in the adult pancreas, such as Ins^low[^ ^8^ ^]^ and Procr⁺ progenitors.^[^ ^37^ ^]^ While post‐mitotic transdifferentiation cannot be entirely excluded,^[^ ^91^, ^92^, ^94^, ^95^ ^]^ the direct derivation of multiple endocrine lineages from a single, proliferative CD168⁺ cell strongly supports genuine multipotency. Together, the multi‐lineage clones we identify point to residual developmental flexibility within adult islet cells.

## 4 Conclusion

In summary, this study identifies CD168 as a conserved surface marker that provides access to the rare, proliferative compartment of pancreatic islets. The development of a *CD168‐CreERT2* genetic model enabled us to delineate a timeline for postnatal β‐cell maturation, revealing a process of gradual functional acquisition coordinated with extensive epigenomic reprogramming. The marker's presence in both murine and human islets, and its strong association with the proliferative fraction of PNETs, highlights its relevance across physiological and pathological contexts. This work positions the CD168⁺ population as a focal point for future strategies aimed at modulating β‐cell mass in diabetes or inhibiting proliferation in neuroendocrine tumors.

## 5 Experimental Section

### Experimental Mice

All animal experiments were approved by the Animal Care and Use Committee of the Center for Excellence in Molecular Cell Science (CEMCS), Chinese Academy of Sciences, with the project license number of IBCB0065 and SIBCB‐S335‐1601‐002‐c4. Both male and female mice with 8–16 weeks were used in this study. Mice were maintained in the CEMCS animal facility under specific pathogen‐free conditions with a 12 h light/dark cycle at room temperature in accordance with the institutional guidelines and ethical regulations, and fed with regular chow and sterilized water by the facility staff.

The *CD168‐CreERT2* knock‐in mouse line was generated by inserting a cassette of CreERT2 in exon 2 of the *CD168 (Hmmr)* gene using CRISPR‐Cas9‐mediated genome editing. gRNA Target Sequence: 5′‐AAAACTTCAGAAGCAACTAAAGG‐3′. A mixture of in vitro‐transcribed Cas9 mRNA, the synthesized gRNA, and the purified donor DNA vector was microinjected into the pronuclei of fertilized C57BL/6J mouse zygotes. The injected zygotes were then implanted into pseudopregnant foster females. The resulting founder (F0) pups were screened for successful insertion of the cassette. Genomic DNA was extracted from tail biopsies. The genotyping protocol is provided in Table S1 (Supporting Information).

For the lineage tracing of CD168^+^ cells, the *CD168‐CreERT2* mice were crossed with *Rosa26‐mTmG* (JAX#7676) reporter mice or *Rosa26‐Confetti* (JAX#017492) reporter strains. In *CD168‐CreERT2;Rosa26‐Confetti* lineage tracing, the dose is 80 mg kg^−1^. This low dose aims to achieve sparse labeling at the expense of labeling efficacy, resulting in each islet containing, on average, only a single labeled cell. This is critical for long‐term clonal analysis, as it ensures that all same‐color progeny within an islet are likely to originate from one founder cell. This allows to accurately track clonal expansion, in line with the method from the previous studies.^[^ ^37^ ^]^

A range of TAM doses (from 20 to 240 mg kg^−1^) and quantified were tested: a) The percentage of islets containing labeled cells. b) The number of independently labeled clones (colors) per islet. It was found that a dose of 80 mg kg^−1^ was the optimal balance: it produced a sufficient number of labeled islets for analysis while ensuring that the vast majority (≈90%) of labeled islets contained only a single Confetti^+^ clone in 8‐week‐old mice. Lower doses yielded too few labeled events, while higher doses (e.g., 240 mg kg^−1^) resulted in multiple clones per islet, which confounds clonal analysis. Therefore, the endpoint that determined 80 mg kg^−1^ as optimal was the achievement of single‐cell, sparse labeling for reliable clonal fate mapping.

In *CD168‐CreERT2;Rosa26‐mTmG* lineage tracing, the dose is 4 mg/25 g (160 mg kg^−1^). This higher dose was used to increase the labeling efficiency to achieve a sufficiently robust signal for downstream ATAC‐seq, RNA‐seq, and GSIS analysis.

For the FACS and immunostaining experiments, the islet cells were isolated from the wild‐type ICR (SLAC) and C57BL/6 (SLAC) mice.

### Patients and Specimens

For the immunostaining of human pancreas sections, a total of 2 normal human donors were randomly collected at Shanghai Ruijin Hospital, with approval from the Human Research Ethics Committee of Ruijin Hospital (approval number: 2025384).

For the immunostaining, FACS, and RNA‐seq analysis of PNETs, a total of 7 PNETs were randomly collected at Shanghai Zhongshan Hospital and The Second Affiliated Hospital, Zhejiang University School of Medicine, between February 2023 and April 2025, with approval from the Human Research Ethics Committee of Zhongshan Hospital (approval number: B2023‐037R). The clinical pathologic diagnosis of PNET cases was determined by pathologists in the Department of Pathology (Table S2, Supporting Information).

### Mouse Islets Enrichment and Purification

Mouse pancreas was perfused by injection of 2–4 mL pre‐cooled (4 °C) digestion buffer (RPMI 1640 medium (Gibco, C11875500) containing 5 mg mL^−1^ type IV collagenase (Worthington, LS 004189, 1000‐1500 U mL^−1^), 5% FBS (fetal bovine serum, EXCELL(SERUM), FSD500), and 1% penicillin‐streptomycin (p/s, Gibco, 15140‐122)) from the common bile duct. Perfusion efficacy was confirmed by uniform tissue distension. The pancreas was then dissected carefully to keep the integrity of the perfused tissue and then incubated at 37 °C for 4 min followed by mechanical dissociation in 37 °C 100 rpm shaker for 16 min. The proper perfusion and digestion time are critical for the islets' yield and structural integrity. Digested tissues were suspended in 20 mL ice‐cold PBS (SparkJade, CR0014) supplemented with 5% FBS and rigorously shaken up and down for 10 times within 20 s, followed by PBS wash for 2 times. The digested tissues were then filtered with 600 µm cell strainers. To enrich islets, the filtered sample underwent density gradient centrifugation or was transferred under the microscope for hand‐picking of islets. To prepare the density gradient centrifugation, the tissue was first re‐suspended in 5 mL histopaque‐1077 (Sigma–Aldrich, 10771) and 6 mL histopaque‐1119 (Sigma–Aldrich, 11191), and carefully overlayed 10 mL PBS onto the suspension without breaking the interface. Then centrifuge at 1114 ×g for 10 min with the slowest acceleration and breaking to obtain the enriched islets compartment at the PBS/histopaque interface. After the initial density gradient centrifugation step, the purity of islets ranged from 20% to 70%, depending on the quality of the earlier perfusion and digestion process. To enhance the purity of islets, the samples were re‐suspended in PBS with 5% FBS for hand‐picking. Islets with high purity were obtained by hand‐picking under a dissecting microscope for at least 3 rounds. Islets were identified by characteristic spherical morphology.

### Immunofluorescence, Whole Mount Staining, and Microscopy

To prepare samples for frozen sections, the dissected pancreas or PNET samples were fixed in 4% paraformaldehyde (PFA, Sigma, P6148) at 4 °C overnight. After 3 times of 10 min PBS washes, tissues were cryoprotected in 30% sucrose/PBS overnight at 4 °C and then embedded in Optimum Cutting Temperature (OCT, Thermo Fisher, D6506). 30 µm‐thick sections were cut using a cryostat (Leica) and stored at −80 °C. Tissue sections were blocked with 10% FBS in PBST (PBS+0.1% Triton X‐100 (Sigma, T8787)) for 1 h at room temperature, then incubated with primary antibodies at 4 °C overnight, followed by washing in PBST, incubation with secondary antibodies and DAPI (Sigma, D9542) for 2 h at room temperature, then sections were washed and mounted for confocal imaging.

To perform whole mount staining, handpicked islets were fixed in 4% PFA at 4 °C for 2 hr, followed by 3 times of PBST washing, then blocked with the whole mount blocking buffer at room temperature for 1 hr. The blocking buffer contained 10% FBS in PBST. Primary antibodies were diluted in blocking buffer and incubated at 4 °C overnight with orbital shaking (25 r min^−1^). After washing with PBST 3 times, the secondary antibodies and DAPI were incubated at 4 °C overnight with orbital shaking (25 r min^−1^). Islets were then washed and mounted with mounting medium for confocal imaging.

All confocal images were captured by the Leica SP8 confocal detection system fitted on a Leica DMI6000 microscope.

All images for a given antibody stain within the same experiment were acquired using identical microscope settings (including laser power, gain, offset, and exposure time). The absolute fluorescence intensity could vary slightly between experimental sessions. To account for this and enable direct comparison, an internal normalization strategy was implemented. Within each individual image, numerous Nkx6.1^high^Ins^high^ cells (representing the mature β‐cell population) were identified and set their average fluorescence intensity as the reference value. The intensity of all other cells (including CD168^+^ cells and their progeny) in that same image was then expressed relative to this internal control. The signals comparable or higher to this average intensity were binned into “high” population, and signals lower than this average intensity were binned into “low” population. This step controls for any minor variations in staining or imaging between samples.

For the YFP, CFP, and GFP reporters from the Confetti system, antibody staining was performed using a Chicken Anti‐GFP antibody (1:500, Invitrogen, A10262), which effectively cross‐reacts with all three variants. For the RFP reporter, its native fluorescence was relied on, which was sufficiently bright for detection. CD168 was detected by Rabbit Anti‐Human/Mouse CD168 (1:50, CST, 87129S) or Rabbit Anti‐Human/Mouse CD168 (1:50, abcam, ab124729) antibody. Nkx6.1 was detected by Rabbit Anti‐Human/Mouse NKX6.1 (1:300, Invitrogen, PA523070) antibody. Insulin was detected by Mouse Anti‐Human/Mouse INS (1:500, Proteintech, 66198‐1‐Ig). Glucagon was detected by Mouse Anti‐Human/Mouse GCG (1:1000, Sigma, G2654). Somatostatin was detected by Rat Anti‐Human/Mouse SST (1:500, Sigma, MAB354). Pancreatic polypeptide was detected by Goat Anti‐Human/Mouse PPY (1:200, Sigma, SAB2500747). Ki67 was detected by Rat Anti‐Human/Mouse Ki67 (1:200, Invitrogen, 14‐5698‐82). The following secondary antibodies in 1:500 dilutions were used: Donkey Anti Chicken Alexa Fluor 488 (Jackson, 703‐546‐155), Donkey Anti Rabbit Alexa Fluor 488 (Invitrogen, A21206), Donkey Anti‐Mouse Alexa Fluor 488 (Invitrogen, A21202), Donkey Anti‐Rat Alexa Fluor 488 (Invitrogen, A21208), Donkey Anti‐Mouse Cy3 (Jackson, 715‐165‐150), Donkey Anti‐Rat Cy3 (Jackson, 712‐165‐150), Donkey Anti‐Rabbit Cy3 (Jackson, 711‐165‐152), Donkey Anti Goat 647 (Invitrogen, A21447), Donkey Anti Mouse Alexa Fluor 647 (Invitrogen, A31571), Donkey Anti‐Rat Alexa Fluor 647 (Invitrogen, A48272) and Donkey Anti‐Rabbit Alexa Fluor 647 (Invitrogen, A31573).

### Preparation of Mouse Islets Single Cell Suspension and FACS Analysis

Purified islet samples were treated with 1 mL Accutase (Thermofisher, 00‐4555‐56) at 37 °C for 4 min, followed by gently pipetting ≈20 times and digestion for another 4 min. CD168 protein can be digested by trypsin,^[^ ^96^ ^]^ so trypsin digestion cannot be used for CD168 immunostaining. The digested cells were filtered through a 70 µm cell strainer (Falcon). For FACS analysis and cell sorting, antibody incubation was performed on ice for 25 min in PBS with 5% FBS at a dilution of 1:200. CD168 antibody (Cell Signaling Technology, 87129S) was incubated on room temperature at a dilution of 1:50, and the second antibody (Donkey Anti‐Rabbit Alexa Fluor 647, Invitrogen, A31573) dilution of 1:100. The following antibodies in 1:200 dilutions were used: eFluor 450 Rat Anti‐Mouse CD31 (Invitrogen, 48‐0311‐82), eFluor 450 Rat Anti‐Mouse CD45 (Invitrogen, 48‐0451‐82), FITC Rat Anti‐Mouse CD326 (EpCAM) (Invitrogen, 11‐5791‐82), PE Rat Anti‐Mouse Ki67 (Invitrogen, 12‐5698‐82), BV421 Mouse Anti‐Human CD56 (BD Pharmingen, 562751). All analysis and sorting experiments were performed using FACS Jazz (Becton Dickinson) and FACS Melody (Becton Dickinson). The purity of the sorted population was routinely checked and ensured to be more than 90%.

The ZIGIR (VitalQuan, 0143) and Ex4‐Cy5 (VitalQuan, 1453) were used for β‐cell enrichment.^[^ ^97^ ^]^ Handpicked islets were incubated with Ex4‐Cy5 (50 nM) at 37 °C for ≈1 h prior to islet dispersion. Mouse islets were dispersed with 1 mL Accutase at 37 °C for 4 + 4 min. The dispersed islet cells were filtered through a 70 µm cell strainer and incubated with 1 µM ZIGIR at 37 °C for 15 min in the cell sorting buffer.

### RNA Isolation and QPCR

RNA isolation of primary cells was performed following the manufacturer's instructions. Samples were lysed in RNAisoplus (Takara, 9108). Extracted RNA was reverse transcribed into cDNA using the Primerscript RT master kit (Takara, DRR036A). qPCR samples were prepared with SYBR Green Mixture (Roche, 04913914001) and detected using the Applied Biosystems Stepone Plus machine. All the qPCR primers were listed in Table S2 (Supporting Information).

### GSIS

About 1500 FACS‐sorted, traced islet cells were used. The cells were cultured on Matrigel (Corning‐Biocoat, 356231) and the experiment was performed following methods previously described.^[^ ^98^ ^]^ The cells were first washed with Krebs buffer 3 times, then pre‐incubated in low glucose (2 mM) for 2 h to remove residual insulin. Subsequently, the cells were washed for 2 times in Krebs buffer followed by incubation in low‐glucose medium for 30 min, and the supernatant samples were collected. Subsequently, the cells were washed 2 times in Krebs buffer followed by incubation in high glucose (20 mM) for 30 min, and the supernatant samples were collected. Subsequently, the cells were washed 2 times in Krebs buffer followed by incubation in KCl (30 mM) for 30 min, and the supernatant samples were collected. Insulin levels contained in the supernatant samples were analyzed using the mouse Ultrasensitive Insulin ELISA kit (Crystal, 90082) following standard protocols.

### Analysis of ScRNA‐seq Data

Filtering, alignment to the mm10 transcriptome, and unique molecular identifier (UMI)‐collapsing were performed using the Cell Ranger (v2) pipeline with default mapping arguments (10X Genomics).

Cell filtering: The Seurat (v3.1.5) R package was used for data integration, analysis, and visualization. To create a Seurat object, genes that were expressed in at least 2 cells and cells that had at least 300 detected genes and 1500 detected UMIs were selected.

Normalization and Scaling: Each dataset was log‐normalized and scaled.

1. Identification of Anchors: The FindIntegrationAnchors() function was used to identify a set of “anchors” between pairs of datasets. This function performed canonical correlation analysis to find mutual nearest neighbors across datasets.
2. PCs selection: The differentially expressed genes were found by the “‘vst”’ method, and the top 2000 differentially expressed genes were selected for PCA analysis. PCs selection was based on an elbow plot. 30 PCs were used for mouse primary pancreatic cells. 40 PCs were used for normal human pancreas and human PNETs.
3. Data Integration: The anchors were then used to integrate the datasets into a single combined matrix using the IntegrateData() function. This step effectively removed technical batch effects while preserving biological variance.

In each analysis, more PCs were also tested for t‐SNE projection, and no changes in cell distribution and clustering were observed.

Dimensional reduction, cell clustering, and data display: Dimensional reduction was performed with the Uniform Manifold Approximation and Projection method. Cell clustering was based on the shared‐nearest neighbor method, and the resolution was set to 2.0 for primary mouse pancreatic cells, normal human pancreas, and human PNETs. Then, clusters were put together as one cell type based on the similarity of the expression profiles and the marker genes. Violin plots, heatmaps, dot plots, and individual UMAP plots for the given genes were generated by using the Seurat toolkit VlnPlot, DoHeatmap, DotPlot, and FeaturePlot functions, respectively.

For mouse pancreas scRNA‐seq analysis, 4 datasets were used:

1. Dataset from Cell 2020:^[^ ^37^ ^]^ Accession number OEP00000249, and all 3 samples OES00014924, OES00014925 and OES00019015 were used.
2. Dataset from Mol Metab 2021:^[^ ^13^ ^]^ Accession number GSE161966, and all 3 samples GSM4928854, GSM4928855 and GSM4928856 were used.
3. Dataset from Diabetologia 2023:^[^ ^39^ ^]^ Accession number GSE203376, and the control diet sample GSM6170636 were used.
4. Dataset from Nat Metab 2023:^[^ ^38^ ^]^ Accession number GSE211799, and all 7 samples GSM6502533, GSM6502534, GSM6502535, GSM6502536, GSM6502537, GSM6502538 and GSM6502539 were used.

For normal human pancreas scRNA‐seq analysis, 1 dataset was used:

1. Dataset from bioRxiv 2025:^[^ ^60^ ^]^ Accession number GSE221156, and 3 healthy (non‐diabetic) samples GSM6846489, GSM6846494 and GSM6846495 were used.

For human PNET scRNA‐seq analysis, 1 dataset was used:

1. Dataset from Int J Biol Sci 2021:^[^ ^61^ ^]^ Accession number GSE162708, and all 2 primary tumor tissue samples GSM4957683 and GSM4957684 were used.

### Preparation of Human PNET Single Cell Suspension

PNET tissues were minced, placed in digestion medium (RPMI 1640 with 5 mg mL^−1^ collagenase IV, 5%FBS, and 1% p/s), and digested for 1 h at 37 °C. After lysis of the red blood cells, a single‐cell suspension was obtained by sequential incubation with Accutase at 37 °C for 10 min with gentle pipetting followed by filtration through 70 µm cell strainers.

### RNA‐seq and Analysis

Total mRNA concentration was determined with NanoDrop ND‐1000, and RNA‐seq libraries were prepared according to the manufacturer's instructions followed by sequencing on Illumina Novaseq‐PE150, which was performed by Novogene company. In total, more than 6 G raw data for each sample were obtained and uniquely mapped to the hg38 human genome with >80% mapping rate for PNET samples and mm10 mouse genome with >70% mapping rate for CD168 lineage tracing samples. Differential gene expression analysis was carried out, and genes with significant alteration were extracted and further analyzed using NovoMagic Bioinformatics Resources.

### ATAC‐seq

ATAC‐seq was performed following the methods previously described.^[^ ^99^ ^]^ FACS‐sorted islet cells were centrifuged at 1000 rpm for 5 min. 1000 cells per sample were suspended in ice‐cold PBS. Cells were pelleted and resuspended in lysis buffer. Following standard protocols using Hyperactive ATAC‐Seq Library Prep Kit for Illumina (Vazyme, TD711‐01). Libraries were sequenced on the Illumina Nova‐seq PE150 platform, which was performed by Annoroad company.

### Analysis of ATAC‐seq Data

The quality control for ATAC‐seq data was conducted using FastQC v0.11.8. Adapters were trimmed using Trim_Galore, and raw FASTQ files were processed accordingly (https://www.bioinformatics.babraham.ac.uk/projects/trim_galore/). Alignment was performed using the Hisat2 v2.1.0^[^ ^100^ ^]^ onto the mouse reference genome mm10, with specified trimming parameters (–trim5 30 –trim3 10). PCR duplicates were removed using the Java package MarkDuplicates.jar.^[^ ^101^ ^]^ ATAC‐seq peaks were identified using the Genrich (https://github.com/jsh58/Genrich) callpeaks function with default parameters. Bigwig tracks were generated with deepTools v3.3.0,^[^ ^102^ ^]^ normalized to RPKM with a binsize of 10 bp. Genomic region signals from ATAC‐seq signals were visualized using deepTools v3.3.0. The GO term enrichment for DEGs was conducted using the Metascape (https://metascape.org).^[^ ^103^ ^]^

### Quantification and Statistical Analysis

Paired or unpaired two‐tailed Student's *t*‐tests were performed when two groups of samples were compared. All the *p*‐values were calculated using GraphPad PRISM 10 with the following significance: n.s., not significant; *p*>0.05; ^*^ *p*< 0.05; ^**^ *p*< 0.01; ^***^ *p*< 0.001; ^****^ *p*< 0.0001. Statistical details for each experiment can be found in the figures and the legends.

## Conflict of Interest

The authors declare no conflict of interest.

## Author Contributions

S.Yuan and Y.A.Z. designed the project. S.Yuan, M.S., and Y.A.Z. wrote the manuscript. S.Yuan performed FACS, immunostaining, qPCR, genetic crosses, genotyping, lineage tracing, and phenotypic analysis. M.S., S.Yuan, C.L., J.C., and Y.T. contributed to bioinformatic data analysis. J.L., X.C., T.X., Z.X., and J.Y. performed FACS and immunostaining. H.F., X.H., T.H., W.W., and L.L., H.B., S.Yan, S.S., A.G., Q.C.Y., and W.S. performed human sample preparation. J.L., A.G., T.X., X.C., Y.T. and J.Y. helped with manuscript editing.

## Supporting information



Supporting Information

## Data Availability

The data that support the findings of this study are available from the corresponding author upon reasonable request.
