# Supplementary material for: CD168 Identifies Proliferating Pancreatic Islet Cells in Murine and Human
Source: Adv Sci (Weinh). 2025 Dec 21;13(10):e10590. doi: 10.1002/advs.202510590 (PMC12915181; doi:10.1002/advs.202510590)
Supplement: Supplementary file 1 — Supporting Information [file ADVS-13-e10590-s001.pdf]

Appendix for:

CD168 Identifies Proliferating Pancreatic Islet Cells in Murine and Human

Shubo Yuan, Jiafu Li, Min Shao, Haili Bao, Ajun Geng, Jialin Yang, Yu Tao, Xinyi Chen, Tianxiong Xiao, Chunye Liu, Zhiyao Xie, Wenqian Song, Qing Cissy Yu, Hongxing Fu, Xu Han, Taochen He, Wenquan Wang, Jianfeng Chen*, Sheng Yan*, Shaohua Song*, Liang Liu*, and Yi Arial Zeng*

Supplementary Figures

Figure S1. ScRNA-Seq unveils CD168$^+$ proliferating immature islet cells.
Figure S2. CD168 marked proliferating immature islet cells in different conditions.
Figure S3. Lineage tracing using *CD168-CreERT2* mouse model.
Figure S4. Multi-omics analysis reveals principles of regulatory process during β cell maturation.

Supplementary Tables

Table S1. Genotyping protocol of *CD168-CreERT2* mouse model.
Table S2. PNET donor information.
Table S3. qPCR primers used in this study.

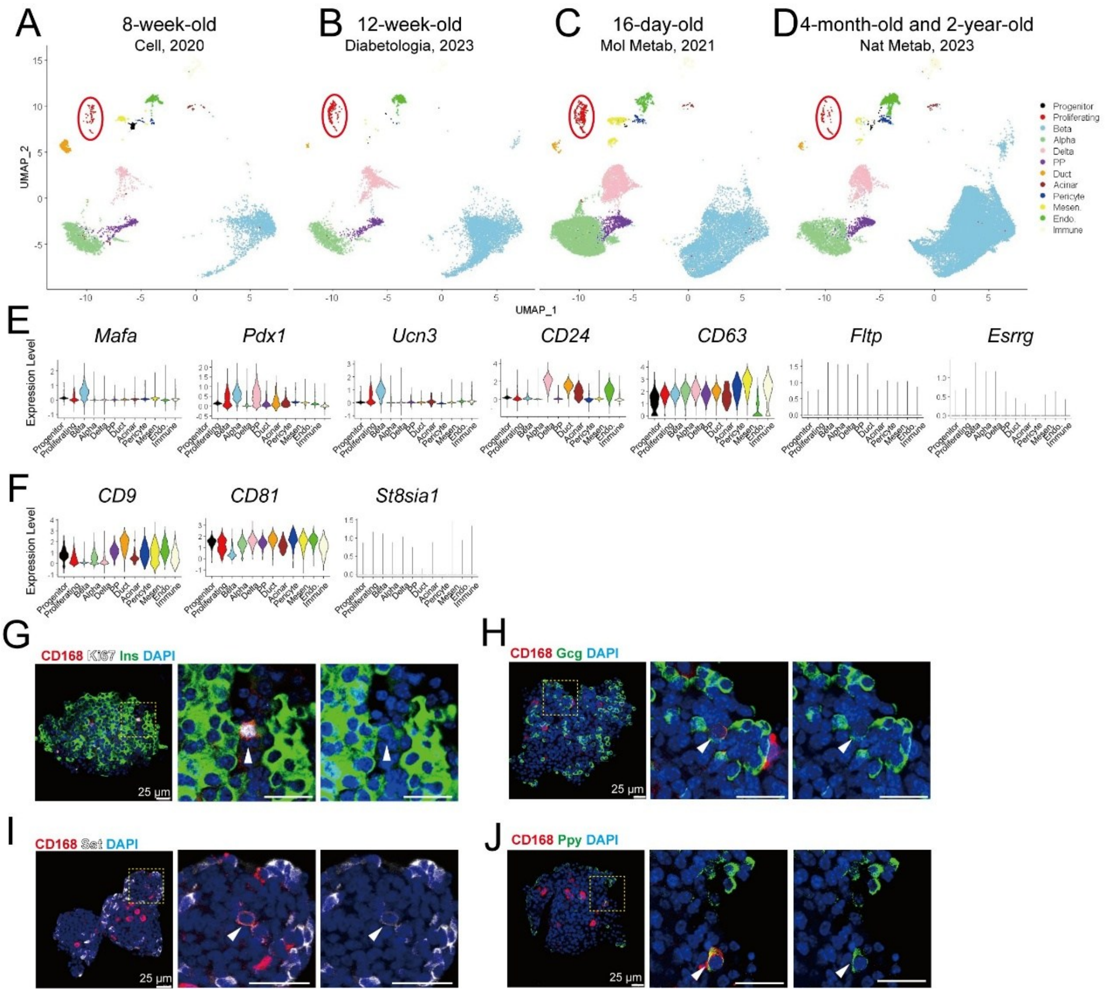

Figure S1. (associated with Figure 1) ScRNA-Seq unveils CD168⁺ proliferating immature islet cells.

A-D) UMAP plot of 68,155 pancreatic single-cell (sc) RNA-seq profiles (points), colored by cluster assignment and annotated post hoc. The new population (Proliferating) is circled in red. The dimplots are splited by datasets. The used datasets were published in Cell 2020 (A), Diabetologia 2023 (B), Mol Metab 2021 (C) and Nat Metab 2023 (D). Mensen., mesenchymal; Endo., endothelial. E-F) The Vln plots show the expression level (log2(TPM+1)) of the indicated gene in each cell type. The proliferating population expressed lower maturation markers (E) and higher immature markers (F) than β cells. The expression levels of *Fltp*, *Esrrg* and *St8sia1* were not detected in the merged datasets. G-J) Representative confocal images of 8-week-old mouse islet whole mount immunostaining, indicating CD168⁺ cells (white solid arrowhead) had lower hormone expression level.

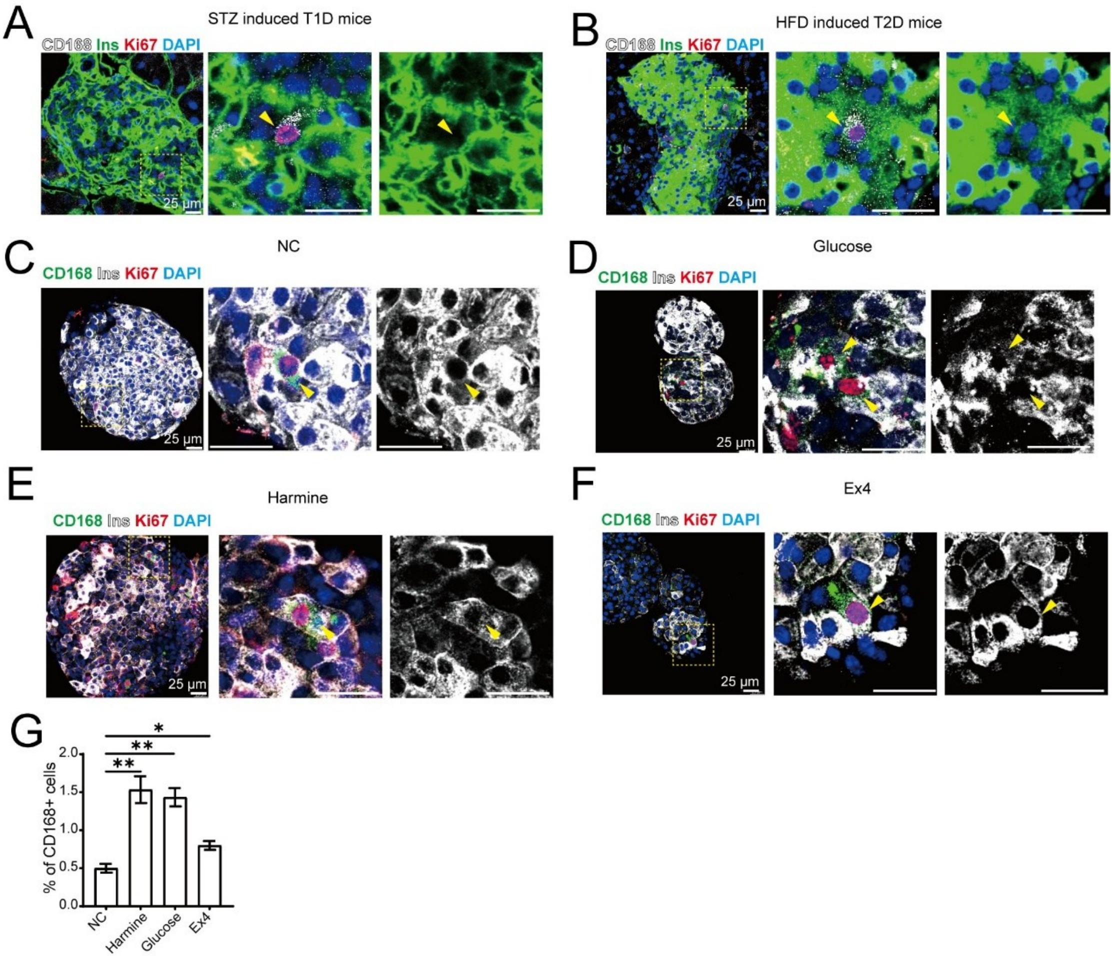

Figure S2. (associated with Figure 2) CD168 marked proliferating immature islet cells in different conditions.

A-B) Representative confocal images of diabetes mouse islet whole mount immunostaining, indicating CD168^{+} cells (yellow solid arrowhead) had lower Insulin expression level and higher Ki67 expression level in streptozotocin-induced type 1 diabetes (T1D) model (The mice were induced with 4 mg/25 g body weight streptozotocin (STZ) injection 2 weeks before harvest. Random-fed blood glucose levels were monitored, and the mice with random blood glucose higher than 20 mM were selected.) (A) and high fat diet-induced type 2 diabetes (T2D) model (The mice were induced with 60 Kcal% fat purified diet 3 months before harvest. Body weight were monitored, and the mice with body weight higher than 40 g were selected.) (B). C-G) Representative confocal images of islet whole mount immunostaining in different stress conditions, indicating CD168^{+} cells (yellow solid arrowhead) had lower insulin expression and higher Ki67 expression in negative control (C), 20 mM glucose stimulation (D), 10 μM Harmine stimulation (E) and 100 nM Exendin-

4 (Ex4) stimulation (F). Isolated mouse islets were incubated in RPMI1640 +10% FBS +1% p/s overnight, and stimulated for 8 hours. Quantification indicating Harmine or glucose or Ex4 treatment increased the proportion of $CD168^+$ cells (G).

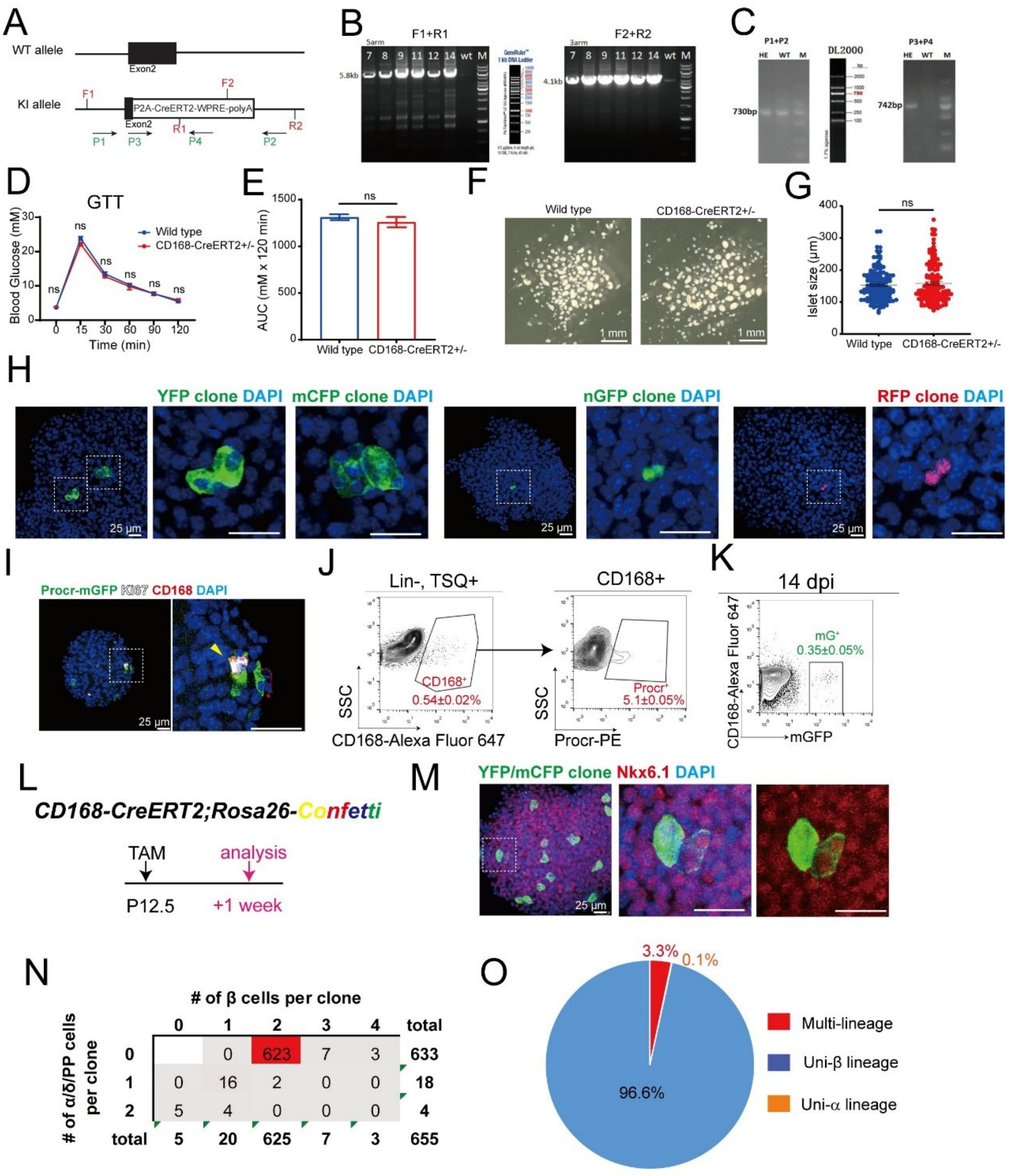

Figure S3. (associated with Figure 3, Figure 4 and Figure 5) Lineage tracing using *CD168-CreERT2* mouse model.

A) Targeting strategy to generate the *CD168-CreERT2* knock-in mouse. Designs of ES clone genotyping primers (red) and mouse genotyping primers (green) are as indicated. B) ES clone genotyping PCR indicating six successful knock-in (KI) clones. C) Genotyping PCR results. D-E) Glucose tolerance test of *CD168-CreERT2* mice and their wild type littermates (D) and the quantification of area under curve I. F-G) Representative images of isolated islets of *CD168-CreERT2* mice and their wild type littermates (F) and the

quantification of islet size (G). H) Representative confocal images of *CD168-CreERT2;Rosa26-Confetti* clones containing all 4 color clones during 8-week long-term tracing. I) Representative confocal images of *Procr-mGFP-2A-lacZ* mouse islets, indicating there were some $CD168^{+}Procr^{+}Ki67^{+}$ cells in islets. J) FACS plots show there were 5.1% $Procr^{+}$ cells in mouse islet $Lin^{-}TSQ^{+}CD168^{+}$ cells. The proportion is similar with multi-lineage percentage. K) FACS plot of *CD168-CreERT2;Rosa26-mTmG* 14 dpi tracing islet cells. L-O) Illustration of *CD168-CreERT2;Rosa26-Confetti* mice. TAM (2 mg/25 g body weight) was injected at pregnancy 12.5 and mice were harvested after 1 week (L). Representative confocal images of islets (M). Quantification of cell number per clone (N). Quantification of clone lineage (O).

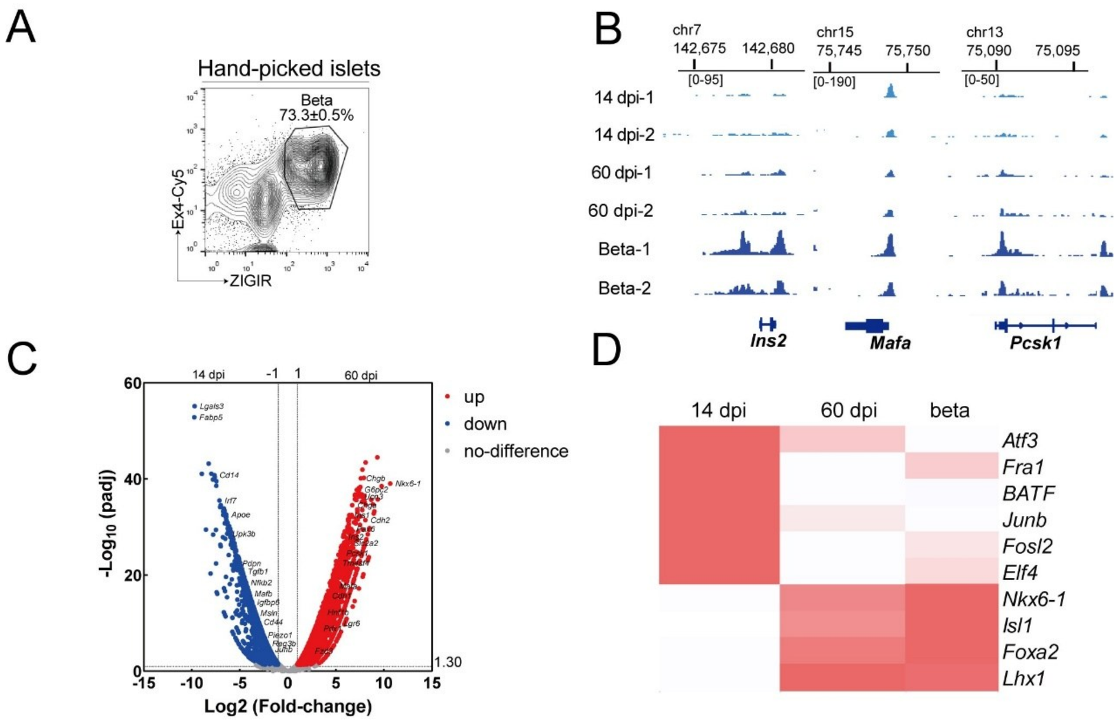

Figure S4. (associated with Figure 6) Multi-omics analysis reveals principles of regulatory process during β cell maturation.

A) FACS of β cells in hand-picked mouse islets, 73.3% ± 0.5% of cells were β cells, the sorted β cells were used for ATAC-seq and RNA-seq. Data were pooled from n=3 biological replicates and presented as mean ± SEM. B) ATAC-seq of 14 dpi, 60 dpi and β cells shows peaks around maturation genes like *Ins2*, *Mafa* and *Pcsk1* were increased with tracing time. C) Volcano plots using RNA-seq demonstrated downregulated expression of islet progenitor signature genes (*Cd14*, *Upk3b*, *Pdpn*, *Msln*, *Piezo1* and *Junb*) and upregulated expression of mature β cell signature genes during the CD168^{+} cell maturation. D) Heatmap of the Fragments per Kilobase Million (FPKM) of novel identified TFs in RNA-seq. Each column represents a sample and each row represents one signature gene. The colors ranging from white to red indicate low to high relative gene expression levels.

Table S1. Genotyping protocol of *CD168-CreERT2* mouse model.

<table>
<tr><td>Primer</td><td colspan="4">Sequence (5’→3’)</td><td>Primer type</td></tr>
<tr><td>P1</td><td colspan="4">GGGCTGGTAGTCTTGGGTTC</td><td>Forward</td></tr>
<tr><td>P2</td><td colspan="4">CTTGCTCGACGTCTCTCGTT</td><td>Reverse</td></tr>
<tr><td>P3</td><td colspan="4">TTGTGGTTGGTGCCATTCCT</td><td>Forward</td></tr>
<tr><td>P4</td><td colspan="4">ATTCAACTTGCACCATGCCG</td><td>Reverse</td></tr>
<tr><td rowspan="19">PCR Reaction System</td><td colspan="4">Reaction Component</td><td>Volume (µl)</td></tr>
<tr><td colspan="4">ddH2O</td><td>14.9</td></tr>
<tr><td colspan="4">10 x Taq PCR Buffer</td><td>2</td></tr>
<tr><td colspan="4">2.5 mM dNTP</td><td>1</td></tr>
<tr><td colspan="4">P1 (10 µM)</td><td>0.5</td></tr>
<tr><td colspan="4">P2 (10 µM)</td><td>0.5</td></tr>
<tr><td colspan="4">Taq DNA Polymerase*</td><td>0.1</td></tr>
<tr><td colspan="4">genomic DNA</td><td>1</td></tr>
<tr><td colspan="4">Total</td><td>20</td></tr>
<tr><td colspan="4"></td><td></td></tr>
<tr><td colspan="4">ddH2O</td><td>14.9</td></tr>
<tr><td colspan="4">10 x Taq PCR Buffer</td><td>2</td></tr>
<tr><td colspan="4">2.5 mM dNTP</td><td>1</td></tr>
<tr><td colspan="4">P3 (10 µM)</td><td>0.5</td></tr>
<tr><td colspan="4">P4 (10 µM)</td><td>0.5</td></tr>
<tr><td colspan="4">Taq DNA Polymerase*</td><td>0.1</td></tr>
<tr><td colspan="4">genomic DNA</td><td>1</td></tr>
<tr><td colspan="4">Total</td><td>20</td></tr>
<tr><td colspan="5">*Taq DNA Polymerase from Takara (Code number: R001A)</td></tr>
<tr><td rowspan="7">Cycling Reaction</td><td>Step</td><td>Temp（℃）</td><td>Time</td><td colspan="2">Note</td></tr>
<tr><td>1</td><td>94</td><td>5 min</td><td colspan="2" rowspan="3"></td></tr>
<tr><td>2</td><td>94</td><td>30 sec</td></tr>
<tr><td>3</td><td>60</td><td>30sec</td></tr>
<tr><td>4</td><td>72</td><td>1 min</td><td colspan="2">repeat steps 2-4 for 35 cycles</td></tr>
<tr><td>5</td><td>72</td><td>5 min</td><td colspan="2" rowspan="2"></td></tr>
<tr><td>6</td><td>12</td><td>Hold</td></tr>
<tr><td>Result</td><td colspan="5">PCR Products:
Mutant: 742 bp; WT: 730 bp.</td></tr>
</table>

	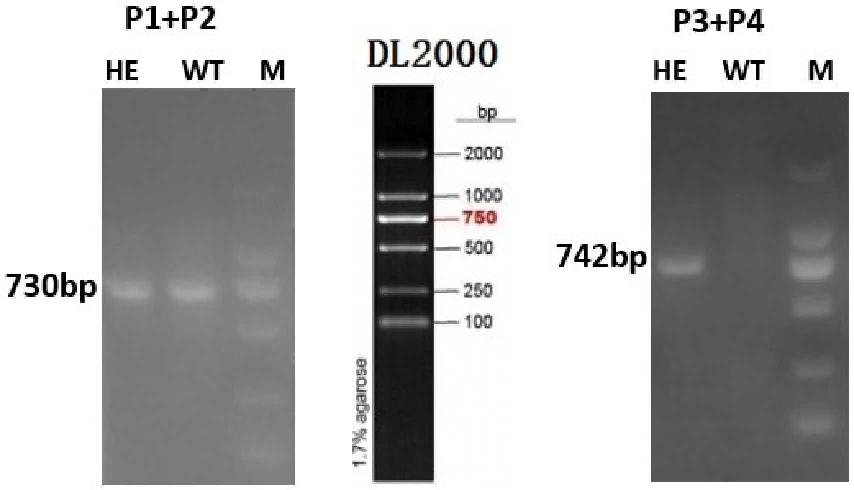 Separated by gel electrophoresis on a 1 % agarose gel.

Table S2. PNET donor information.

Donor ID	Sample ID	Gender	Age (years)	KI67 Index	Pathology
1	#Hu240906	male	72	3%	G2
2	#Hu240813	male	69	2%	G1
3	#Hu241129	female	36	1%	G1
4	#Hu241227	male	46	2%	G2
5	#Hu250106	male	67	10%	G2
6	#Hu250421	female	56	1%	G1
7	#Hu230213	female	81	3%	G2

Table S3. qPCR primers used in this study.

Mouse qPCR primers

Gene	Forward primer	Reverse primer
CD168	ACTTCAGAAGCAACTAAAGGACC	AGCAAGCAAGGTTGTATCTTTGT
Hprt	TCAGTCAACGGGGGACATAAA	GGGGCTGTACTGCTTAACCAG
Ins1	TGGCTTCTTCTACACACCCAAG	ACAATGCCACGCTTCTGCC
Mki67	ATCATTGACCGCTCCTTTAGGT	GCTCGCCTTGATGGTTCCT
Nkx6.1	CTGCACAGTATGGCCGAGATG	CCGGGTTATGTGAGCCCAA

Human qPCR primers

Gene	Forward primer	Reverse primer
CD168	AACAAGCTGAAAGGCTGGTCA	GGGTATGAGCAGCACTACTTTT
HPRT	GCTATAAATTCTTTGCTGACCTGCTG	AATTACTTTTATGTCCCCTGTTGACTGG
MKI67	GCCTGCTCGACCCTACAGA	GCTTGTCAACTGCGGTTGC
CENPE	GCATCGCAAAGCCAAGGATTC	TCTTGAAGTCTGTTGGTCAACTC
INS	GCAGCCTTTGTGAACCAACAC	CCCCGCACACTAGGTAGAGA
NKX6.1	AGGGCTCGTTTGGCCTATTC	AGAGGCTTATTGTAGTCGTCGT
UCN3	CCCACAAGTTCTACAAAGCCA	TCCCGAAGAGGCGTCTCTG
